# Short-wavelength sensitive opsin (SWS1) as a new marker for vertebrate phylogenetics

**DOI:** 10.1186/1471-2148-6-97

**Published:** 2006-11-15

**Authors:** Ilke van Hazel, Francesco Santini, Johannes Müller, Belinda SW Chang

**Affiliations:** 1 Departments of Ecology & Evolutionary Biology, Cell & Systems Biology, and Centre for the Analysis of Genome Evolution & Function, University of Toronto, 25 Harbord Street, Toronto, Ontario, M5S3G5, Canada; 2 Current address: Humboldt-Universität zu Berlin, Museum für Naturkunde, 10099 Berlin, Germany

## Abstract

**Background:**

Vertebrate SWS1 visual pigments mediate visual transduction in response to light at short wavelengths. Due to their importance in vision, SWS1 genes have been isolated from a surprisingly wide range of vertebrates, including lampreys, teleosts, amphibians, reptiles, birds, and mammals. The SWS1 genes exhibit many of the characteristics of genes typically targeted for phylogenetic analyses. This study investigates both the utility of SWS1 as a marker for inferring vertebrate phylogenetic relationships, and the characteristics of the gene that contribute to its phylogenetic utility.

**Results:**

Phylogenetic analyses of vertebrate SWS1 genes produced topologies that were remarkably congruent with generally accepted hypotheses of vertebrate evolution at both higher and lower taxonomic levels. The few exceptions were generally associated with areas of poor taxonomic sampling, or relationships that have been difficult to resolve using other molecular markers. The SWS1 data set was characterized by a substantial amount of among-site rate variation, and a relatively unskewed substitution rate matrix, even when the data were partitioned into different codon sites and individual taxonomic groups. Although there were nucleotide biases in some groups at third positions, these biases were not convergent across different taxonomic groups.

**Conclusion:**

Our results suggest that SWS1 may be a good marker for vertebrate phylogenetics due to the variable yet consistent patterns of sequence evolution exhibited across fairly wide taxonomic groups. This may result from constraints imposed by the functional role of SWS1 pigments in visual transduction.

## Background

Opsins, or visual pigments, form the first step in the visual transduction cascade in the photoreceptor cells of the retina. By means of a covalently-bound retinal chromophore, opsins are able to respond to light by changing conformation, which activates a second messenger G-protein, and triggers a biochemical cascade that eventually results in a neural signal to the brain that light has been perceived [1]. Opsins are a member of the extremely large superfamily of integral membrane G-protein coupled receptors (GPCR's), with thousands of genes present in the human genome alone [2]. This family is involved in a diverse array of physiological functions in vertebrates, including neurotransmission, learning, memory, and various endocrine and hormonal pathways. All of its members are thought to share the same tertiary structure, mechanisms of activation, and activation of G proteins, even if the downstream effectors of the G proteins may differ. Despite the vast array of functions mediated by this family of receptors, the highly conserved seven helical transmembrane structure of GPCR's as a whole (particularly the Class A type, of which opsins are a member) has ensured that insertions and deletions remain rare, particularly in transmembrane regions.

Visual pigments can vary widely in their wavelength of maximal absorption, ranging from the ultraviolet to the red. The molecular basis of spectral sensitivity depends on interactions between amino acids within the binding pocket of an opsin protein and its associated light-sensitive chromophore. Any variation in the amino acid sequence of a given opsin can, therefore, directly influence the spectral wavelengths an organism can detect. Phylogenetically, visual pigments are divided into 5 groups, roughly reflecting their function in vision, such as whether they are active during the day (cone opsins) or at night (rod opsins), and the spectral tuning of the wavelengths at which they are maximally sensitive [3-5]: red/green or long-wavelength sensitive cone opsins (LWS; approx. 500–570 nm), rod-like or medium-wavelength sensitive cone opsins (RH2; approx. 465–520 nm), ultraviolet/violet or short-wavelength sensitive type 1 cone opsins (SWS1; approx. 360–430 nm), blue or short-wavelength sensitive type 2 cone opsins (SWS2; approx. 430–460 nm), and the rod opsins active at low light levels (RH1; approx. 500 nm). The SWS1 opsins are the shortest wavelength sensitive opsins, and are generally expressed in a particular type of cone photoreceptor found throughout vertebrates that is characterized by an extremely short outer segment [6], though exceptions do exist [7]. Only a few types of vertebrates, such as those living in extreme low light environments (subterranean or deep sea habitats) are thought to lack this type of cone. For example, pseudogenes have been identified in the blind Ehrenberg's mole rat [8], as well in the bottle-nosed dolphin [9] and a number of whales [10]. Similarly, organisms with primarily nocturnal behaviours may also lack the SWS1 opsin; such as the owl monkey and the bushbaby [11].

**Figure 1:**
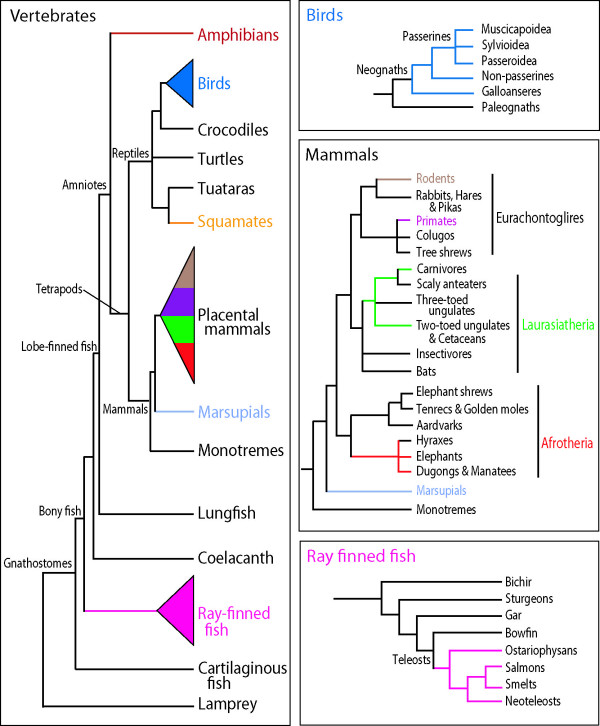
Summary of vertebrate evolutionary relationships, based on morphological and molecular data [18, 32-39]. Colours indicate taxonomic groups represented in the SWS1 data set.

Perhaps due to the highly conserved nature of its role in vertebrate vision, the SWS1 opsin (hereafter SWS1) occurs as a single copy nuclear gene in almost all animals investigated thus far. However, despite the fundamental importance of this gene for vision at short wavelengths, SWS1 exhibits considerable sequence variation across the diversity of vertebrates that have been investigated. This variation may be a product of SWS1 functional diversity, as measured by absorption sensitivities [12-14], which in visual pigments have often been found to be optimized to specific visual environments [5,15,16]. To investigate the molecular evolution of SWS1, we conducted phylogenetic analyses of the gene using available vertebrate sequences. SWS1 genes have been cloned from a considerable variety of vertebrates, ranging from the lamprey to mammals. Surprisingly, we found that this single gene appears to reconstruct many of the commonly accepted relationships among vertebrates (Figure 1), for both deeper and more recent divergences. Indeed, SWS1 results were comparable to those obtained from more exhaustive analyses using multi-gene data sets [e.g. [17-20]]. Here, we present a comprehensive phylogenetic analysis of vertebrate SWS1 sequences. We then investigate the characteristics of this gene that contribute to its evident success as a phylogenetic marker across a broad taxonomic range.

## Results

### Phylogenetic analyses

**Figure 2:**
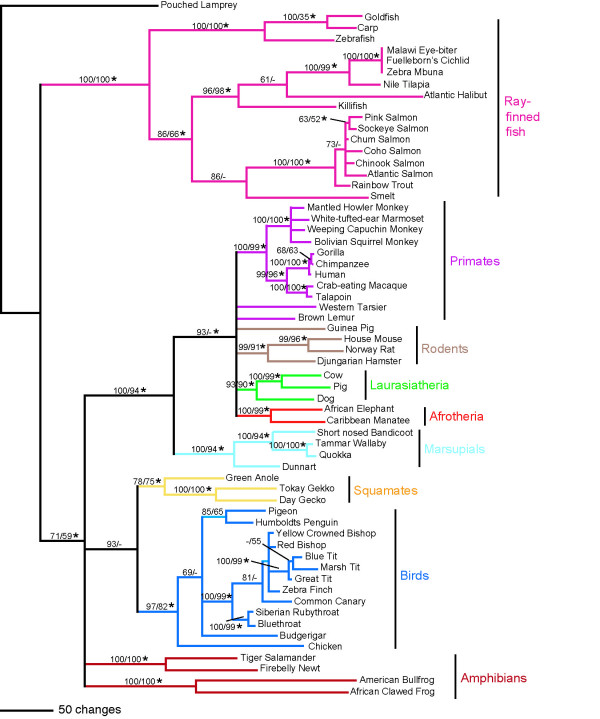
Maximum parsimony phylogeny. Strict consensus of 432 equally most parsimonious trees (length = 3965, CI = 0.35, RI = 0.75) found in a heuristic search with 10,000 replicates. Bootstrap percentages above 50% for MP analyses (1000 replicates), followed by those for ML analyses under the GTR+I+Γ model (100 replicates) are indicated above the nodes. Dashes represent less than 50% bootstrap support. An asterix denotes a posterior probability of ≥0.95 in the Bayesian analysis. Colours correspond to vertebrate groups as indicated in Figure 1.

Sixty-two vertebrate SWS1 opsin nucleotide coding sequences were obtained from GenBank, aligned using ClustalX [21], and analyzed using a variety of phylogenetic methods including maximum parsimony [22,23], maximum likelihood [24,25], and Bayesian methods [26]. A series of nested likelihood ratio tests were performed using ModelTest [27] in order to determine which nucleotide model of those tested best fit the data. Of the nucleotide models commonly implemented for phylogenetic analysis, the general time-reversible model incorporating parameters for invariant sites, as well as among-site rate heterogeneity (GTR+I+Γ) [28-30] was found to fit the data significantly better than any simpler model. This model was therefore used in subsequent likelihood and Bayesian phylogenetic analyses. Assessing confidence in nodes of the phylogeny was accomplished by bootstrap analysis [31] or Bayesian posterior probabilities [26]. The results of the phylogenetic analyses are shown in Figure 2, with the bootstrap values of the maximum parsimony (MP), maximum likelihood (ML) and posterior probabilities of the Bayesian analyses mapped onto the MP tree.

In all analyses, the reconstructed clades were remarkably similar to currently accepted vertebrate relationships based on morphological and molecular analyses (Figure 1). Among the available sequences, however, there are quite a few groups not represented in our dataset, such as cartilaginous fish, monotremes, turtles, crocodiles and snakes. The lack of adequate sampling is particularly evident in the non-tetrapod vertebrates, with the only sequences available being some of the more recently derived ray-finned fish lineages. However, on the basis of the taxa available, the vertebrate clade is divided into two major groups: actinopterygians (ray-finned fishes), and tetrapods. The latter clade is further divided into modern amphibians (frogs and salamanders), mammals, and reptiles (birds+squamates). This is in surprisingly good agreement with the generally accepted relationships among the major vertebrate lineages according to molecular and morphological data as summarized in Figure 1[18,32-39].

Within the ray-finned fishes, our trees support the basal position of ostariophysans (carp, goldfish, and zebrafish), followed by the neoteleosts, salmonids, and smelt, a situation congruent with morphological [40], mitochondrial [35] and nuclear data [41]. Between the latter three clades, however, the relationships remain debated: in some morphological studies salmonids and smelts form a clade [42], whereas in other investigations salmonids group with the neoteleosts [40]. A monophyly of smelts and neoteleosts has also been proposed [43,44], and has since received support from molecular analyses [20,35]. Our analyses are in agreement with the foremost hypothesis, having salmonids and smelts as a monophyletic sister group to neoteleosts.

In our analyses, there is only weak support for the monophyletic grouping of modern amphibians (30% MP bootstrap, 24% ML bootstrap, 0.66 Bayesian posterior probability). The paraphyly of amphibians has been suggested by Carroll [45] using morphological and paleontological data; by contrast, most research, including the remaining paleontological studies and all molecular analyses, disagree with this hypothesis and maintain the monophyly of modern amphibians [46,47]. It should be mentioned, however, that only four amphibian sequences were available for this study, which might be the reason for the lack of resolution in our results.

Within birds, our results confirm the chicken as basal and sister to the Neoaves (all other birds), with the passerines (Passerida) as monophyletic and most derived, a result congruent with recent molecular studies [37,48]. Previous studies by Sibley and Ahlquist [48] using DNA-DNA hybridization, which since has been supported by nuclear and mitochondrial sequence data [38,49], divided Passerida into three major clades: Muscicapoidea (represented in our phylogeny by the bluethroat and Siberian rubythroat), Sylvoidea (tits), and Passeroidea (finch and bishops); patterns of these relationships were identified in all our reconstructed phylogenies.

Among mammals, marsupials are found to be the basal lineage within Mammalia, followed by the monophyletic groupings of rodents (minus the guinea pig), laurasiatherians, afrotherians, and higher primates; these relationships are all well supported by recent research [see [50] for review]. Moreover, the more recent divergences within these mammalian clades also resemble the results found by other investigations. For example, all three phylogenies show apes and Old World monkeys to be monophyletic, forming the catarrhines, with the New World monkeys, or platyrrhines, as a sister group, which together form the higher primates. Although their positions are unresolved in the MP consensus tree, there is some support for the prosimians (the lemur and tarsier) as the most basally positioned primates in the bootstrap (57% ML, 48% MP) and Bayesian analyses (0.9 posterior probability). Our results correspond not only with molecular phylogenies constructed using nuclear [51] as well as mitochondrial [52] datasets, but also with morphological data [53].

### Patterns of sequence variation in the SWS1 data set

Given the utility of SWS1 for elucidating vertebrate evolutionary relationships across a range of divergences, we explored patterns of sequence variation in our data set by estimating parameters such as base composition, among-site rate heterogeneity, and informative sites using parsimony and likelihood phylogenetic methods, and then compared them to those published for other molecular phylogenetic data sets.

The aligned SWS1 nucleotide dataset consisted of 1083 characters, of which 686 (63%) were parsimony informative. The proportion of invariant sites in our data set was estimated in two ways: (1) by calculating the observed number of invariant sites in our alignment, and (2) by estimating the number of sites likely to be invariant under a particular model of evolution (Table 1). Within vertebrates, there were a relatively small proportion of sites estimated as invariant (I = 0.17). Within the data partitions corresponding to the major vertebrate groups, ML estimates do not exceed 0.34. SWS1 tended to have similar proportions of invariant sites as other nuclear markers commonly used for phylogenetic purposes, as estimated using ML methods, for example RAG1 (0.34, squamates [54], 0.36, amphibians [55], 0.40, rodents [56]) or RAG2 (0.24, frogs [55]). While it is generally assumed that a lower proportion of invariable sites might be indicative of greater phylogenetic information in the data set, this parameter is often difficult to estimate accurately [57,58].

**Table 1:** Substitution frequencies and rate heterogeneity parameters

		**A↔C**	**A↔G**	**A↔T**	**C↔G**	**C↔T**^a^	α^b^	**I (ML)**^c^	**I**^d^
**All sites**	Fish	0.778	2.419	1.328	0.984	3.192	1.203	0.262	0.412
	Reptiles	1.081	4.598	0.907	3.330	2.516	0.392	0.000	0.611
	Mammals	1.322	4.876	0.838	0.727	4.672	1.473	0.345	0.530
	Tetrapods	1.091	4.120	0.969	1.431	3.341	1.029	0.244	0.367
	Vertebrates	0.987	3.326	1.084	1.298	3.115	1.014	0.166	0.263
									
**Codon #1**	Fish	2.338	2.645	1.434	0.793	2.877	0.422	0.000	0.334
	Reptiles	2.383	2.148	0.777	0.579	1.313	0.312	0.000	0.629
	Mammals	2.504	4.097	0.739	0.828	3.515	1.445	0.447	0.646
	Tetrapods	2.051	2.781	0.935	0.602	1.891	0.780	0.212	0.457
	Vertebrates	2.188	2.473	1.008	0.741	2.256	0.837	0.122	0.319
									
**Codon #2**	Fish	5.561	9.417	3.878	23.904	10.450	0.411	0.000	0.629
	Reptiles	0.411	7.219	0.549	5.696	3.547	1.936	0.666	0.806
	Mammals	1.654	11.198	2.489	6.140	7.073	0.364	0.298	0.747
	Tetrapods	1.727	8.563	1.226	6.564	4.313	0.403	0.119	0.593
	Vertebrates	2.435	7.089	1.480	7.343	4.679	0.647	0.108	0.435
									
**Codon #3**	Fish	0.454	2.419	1.878	0.286	3.282	3.877	0.013	0.103
	Reptiles	0.377	4.589	0.000	0.431	4.089	1.002	0.000	0.343
	Mammals	0.985	5.023	0.810	0.194	4.248	2.907	0.000	0.197
	Tetrapods	0.623	4.283	0.973	0.679	3.599	3.058	0.028	0.050
	Vertebrates	0.521	3.386	1.169	0.622	3.027	4.665	0.032	0.036

^a ^Parameters are reversible and measured against the G↔T parameter with a value of 1

^b ^Shape parameter for the gamma distribution.

^c ^Proportion of invariant sites estimated using maximum likelihood methods

^d ^Proportion of invariant sites calculated from raw alignment data using MEGA3 [131].

The parameter (α) describing the shape of the Γ-distribution used to account for among-site rate heterogeneity was estimated for the SWS1 data set using likelihood methods to be approximately 1.0 (Table 1). This suggests a fairly even distribution of different substitution rates across sites. Other nuclear genes widely used in phylogenetic analyses tend to have α estimates of at least 1. For example, RAG1 ranges from 1.0 in amphibians [55] to 1.7 in squamates [54]. When α is equal to 1, substitution rates are exponentially distributed, which is intermediate between the bell-shaped curves at higher values (α > 1) and 'L' shaped functions at lower values (α < 1). This exponential shape suggests that there is a more evenly distributed range of substitution rates across sites than would be the case for higher or lower α values. It has been suggested that higher values of α might be better in aiding phylogenetic resolution [59]. But, a more even range of slow to fast evolving sites may be best for phylogenetic analyses because it incorporates both slow sites to carry a signal from deeper divergences, as well as fast sites for more recent divergences. Past studies, based on both empirical data [60] and simulations [59,61] have suggested that large amounts of among-site rate variation (as indicated by low values of α) such as those often found in some mitochondrial data sets, might tend to adversely affect phylogenetic signal.

Furthermore, the variability observed in SWS1 does not appear to be concentrated on third base positions only, as is often the case in many genes; relative to other molecular markers a great deal of variation is found at first and second positions as well. First, although about 92% of third codon positions were parsimony informative, the proportion of informative sites present in first and second codon positions was 55% and 41%, respectively. Second, estimates of invariant sites (I) were generally low across all three codon positions (Table 1), though the proportion of invariant sites was lowest at third positions, as expected. Third, relatively high α values were also found for first (0.84) and second codon positions (0.65), though highest at third positions (4.7).

Most protein-coding data sets show higher α values (and lower I values) at third codon positions compared to first and second positions [62]. However, the values of α at first and second codon positions in the SWS1 data set were comparatively high. For example, under a similar model of evolution (GTR+I+Γ), Dettai and Lecointre [63] estimated α values of 0.29 for both codon positions in a portion of the MLL gene in fish, as compared with 0.42 and 0.41 for first and second positions in fish SWS1 genes. This would suggest that variation in substitution rates tends to be more evenly distributed across codon positions in the SWS1 data set, and that more sites in the gene are phylogenetically informative.

Maximum likelihood estimates of substitution rate parameters in the SWS1 data set under the GTR+I+Γ model did not exhibit substantial skew when estimated across vertebrates, with values ranging only from about 1.0 to 3.3 (Table 1). This range of values is smaller than many other data sets, including cyt b in birds (0.5 to 8.8 [37]), RAG1 in squamates (1.0 to 7.6 [64]), and RAG1 in amphibians (1.0 to 5.7 [55]). A skewed rate matrix may decrease the number of states a given site can have, resulting in increased homoplasy and potential loss of phylogenetic information. Although this has yet to be investigated in detail in many data sets, a recent comparison of mitochondrial and nuclear genes in insects [65] found that nuclear genes tended to have more homogeneous patterns of among-site rate variation (*i.e.*, larger α values), as well as more symmetrical transformation rate matrices, and that these patterns appeared to be associated with phylogenetic utility in their data sets.

Furthermore, substitution rate matrices were estimated in different data partitions of the SWS1 data set corresponding to the different vertebrate groups and/or codon position (Table 1). Overall, the rate matrices remain relatively homogeneous and unskewed across different partitions of the data set, although there were certain partitions exhibiting differences in particular rate parameters. For example, there is some variation in substitution rates between C and G in reptiles, as compared with fish and mammals (3.3, 0.1, and 0.73 respectively), and across vertebrates at second codon positions, as compared with first and third positions (7.3, 0.7, and 0.6 respectively). Also, fish generally tend to have higher substitution rates relative to other vertebrate groups at second codon positions (Table 1).

Base composition was estimated using likelihood methods under the GTR+I+Γ model for the complete data set, as well as within partitions of the data corresponding to individual codon positions, and the major vertebrate groups (Table 2). Base compositional biases can be problematic in phylogenetic analyses, particularly if there is convergence in bias across unrelated groups [66-68]. The SWS1 data set does not appear to be affected in this manner. Despite a significantly heterogeneous base compositional bias overall (chi-square test of homogeneity *p *< 0.001, df = 183; Table 2), in the different data partitions, corresponding to the major vertebrate groups and/or codon position, the base frequencies are generally found to be homogeneously distributed throughout the data set, except in tetrapods and in third positions of many vertebrate groups (Table 2). Second positions showed a slightly increased frequency of T, but this was also found to be homogeneous across the data set (Table 2). The high frequency of T at second codon positions is also seen in genes such as rod opsin and cyt b, and presumably reflects a strong functional constraint in genes coding for transmembrane proteins, which contain many hydrophobic amino acids such as Ile (ATY), Phe (TYY) and, Leu (YTR) [69,70]

**Table 2:** Base composition and χ2 tests of homogeneity

		**A**	**C**	**G**	**T**	**Avg # bps**	**χ2**	**df**	***p***
**All sites**	Fish	0.214	0.290	0.249	0.247	1008	40.53	48	0.770
	Reptiles	0.165	0.344	0.263	0.228	932.7	18.77	45	1.000
	Mammals	0.191	0.293	0.246	0.271	1029	36.98	69	0.999
	Tetrapods	0.197	0.315	0.242	0.246	995.4	419.3	129	**<<0.001**
	Vertebrates	0.210	0.306	0.239	0.245	999.6	613.4	183	**<<0.001**
									
**Codon #1**	Fish	0.266	0.160	0.289	0.286	336.0	7.590	48	1.000
	Reptiles	0.235	0.198	0.295	0.271	310.9	8.498	45	1.000
	Mammals	0.242	0.205	0.282	0.272	342.9	12.94	69	1.000
	Tetrapods	0.260	0.198	0.279	0.263	331.8	37.29	129	1.000
	Vertebrates	0.281	0.171	0.279	0.269	333.2	97.57	183	1.000
									
**Codon #2**	Fish	0.230	0.262	0.163	0.345	336.0	3.147	48	1.000
	Reptiles	0.220	0.244	0.184	0.352	310.9	10.32	45	1.000
	Mammals	0.221	0.235	0.184	0.359	342.9	5.601	69	1.000
	Tetrapods	0.222	0.244	0.179	0.356	331.8	18.94	129	1.000
	Vertebrates	0.224	0.250	0.176	0.349	333.2	76.50	183	1.000
									
**Codon #3**	Fish	0.143	0.392	0.280	0.186	336.6	84.81	48	**0.001**
	Reptiles	0.050	0.588	0.311	0.051	310.8	92.56	45	**<<0.001**
	Mammals	0.118	0.416	0.266	0.200	342.9	66.27	69	0.571
	Tetrapods	0.129	0.454	0.253	0.164	331.7	1363.3	129	**<<0.001**
	Vertebrates	0.143	0.432	0.245	0.179	333.1	1678.6	183	**<<0.001**

In summary, molecular patterns in the vertebrate SWS1 data suggest a substantial amount of variation across the three codon positions, as well as high among-site rate variation throughout the gene. As well, the substitution rates tend to be fairly homogeneous among the different classes. Although there is some bias in nucleotide composition across different codon positions, this bias appears to be non-convergent.

## Discussion

Despite the ever increasing number of gene sequences available in the databases, it remains surprisingly difficult to select genes that will be useful for phylogenetic analyses, particularly across a variety of taxonomic ranges. Known issues in molecular phylogenetics such as model mis-specification, gene paralogs and alignment ambiguities often contribute to misleading results [71], and accounting for effects such as among-site rate heterogeneity can substantially alter results. For example, early analyses of mtDNA data tended to place the root of the avian phylogeny within passerines [72-75], a result in strong conflict with prior phylogenetic studies [48]. Further analysis of mitochondrial data that accounted for unequal evolutionary rates among sites, however, recovered the traditional division of birds between palaeognathans and neognathans, with passerines being a phylogenetically derived neognath lineage [76]. Similarly, correcting for base compositional bias in a mammalian data set of full mitochondrial genomes increased support for marsupials as the sister group of placentals [34], as opposed to the original analysis, which supported a marsupial-monotreme grouping [77]. Many characteristics have been identified as problematic; however, we know little about exactly what characteristics make a strong molecular marker. For example, in insects, comparisons of nucleotide substitution patterns and phylogenetic utility between nuclear ribosomal and protein coding genes [78] as well as between nuclear and mitochondrial genes [65] identified a number of features as useful for phylogenetic analyses, including larger values of α (parameter for among-site rate heterogeneity), and relatively unskewed substitution rate parameters. However, such studies remain relatively rare in the literature.

Mitochondrial genes have been widely used in molecular systematics due to the relative ease with which mitochondrial genes, or even whole genomes can be amplified and sequenced, as well as the absence of problematic features often associated with nuclear markers such as introns, heterozygosity, and paralogy. Mitochondrial genes can, however, suffer from some marked disadvantages. In most cases mitochondrial genes are thought to evolve much faster than nuclear genes [[79], but see [80]], and they may be subject to significant rate accelerations and decelerations in evolutionary history [81], possibly due to changes in factors such as metabolic rate [82]. Such rate differences can easily lead to positively misleading topological effects [83], and may be particularly problematic in resolving deeper relationships or rapid radiations such as those found within mammals [84]. Furthermore, mitochondrial data sets can often be biased in terms of base composition, which has been found to contribute to misleading signal in a number of data sets including basal vertebrates [66,85], birds [76] and mammals [34,86,87]. However, their faster rate of evolution in comparison to nuclear genes can be useful for resolving more recent relationships [65].

Recent years have shown a dramatic increase in the number of nuclear genes that have been developed for vertebrate phylogenetics in order to complement and expand the set of useful phylogenetic markers [88-90]. Some nuclear genes have been used with success in multiple vertebrate groups ranging from fish to mammals, for example RAG1 and 2 [91-93], c-mos [37,64], c-myc [38,56], MLL [41,63], and 18S [94,95]. A host of other nuclear genes have been used primarily in particular vertebrate groups, such as rod opsin in ray-finned fish [63,69], and more recently amphibians [55,96]; β-fibrinogen introns in birds [97], and a variety of genes aimed at resolving higher level mammalian phylogenetics: IRBP, α-2B adrenergic receptor, aquaporin, β-basein, γ-fibrinogen, κ-casein, protamine, and von Willebrand Factor [84].

Despite the success of nuclear markers in resolving some of the longstanding problems in vertebrate phylogenetics [32,50], several issues continue to plague many molecular data sets. First, alignment issues, though long recognized as problematic, often tend to be overlooked in many data sets. Some sequences, particularly ribosomal genes such as 18S, are known to be difficult to align properly [98], and these alignment ambiguities can significantly affect phylogeny reconstruction [95,99]. However, these problems can easily be avoided by careful selection of molecular markers that are unambiguously alignable. Second, and even more importantly, some of the important assumptions currently incorporated into commonly used phylogenetic methods may not be reasonable for many molecular data sets. For instance, most molecular models of evolution assume that state frequencies, and even more importantly, substitution rate frequencies do not change over evolutionary time, assumptions incorporated in likelihood/Bayesian methods which tend to model molecular evolution as stationary, homogeneous Markov processes [100].

Even though nuclear data sets tend to suffer fewer problems than mitochondrial genes with respect to base compositional changes across a phylogeny, there are examples of nuclear data sets for which nonstationarity can yield positively misleading results, if there is convergence in base compositional bias [101,102], or worse yet, codon bias [66,103] among lineages. The use of phylogenetic methods that have been developed to take into account nonstationarity in base frequencies using either distance [67,104] or likelihood approaches [105] can at least somewhat ameliorate these effects, though not for codon bias issues [66,103].

More recently, the use of genome-based approaches has enabled more extensive investigations of sources of systematic bias, or inconsistency in phylogenetic analyses [102,106-108] and identified new effects difficult to detect in smaller data sets, such as site-specific changes in evolutionary rates among lineages, or heterotachy [109-111]. However, these issues are only just being addressed, and the robustness of current phylogenetic models to such violations explored [112-116]. Similarly, the issue of changes in substitution rate frequencies across a phylogeny, or nonhomogeneity, has received relatively little attention, though it has been recognized as a potential problem [67,104,117,118]. Accounting for such effects in more complex models of evolution may be useful for genomic scale analyses, but it is not clear how much power such parameter-rich models may have for relatively small data sets. Selecting genes less susceptible to these effects would tend to obviate the necessity of implementing more complex models, and therefore tend to increase the statistical power of likelihood and Bayesian phylogenetic methods.

With regard to some of the issues plaguing many molecular data sets, SWS1 visual pigment genes offer several clear advantages. First, this gene has very few indels in its evolution across vertebrates, making for a largely unambiguous alignment, and it is a single copy nuclear gene with no paralogs of high sequence similarity. Second, for the vertebrate SWS1 data set, base frequencies were found to be fairly constant across the phylogeny. There was little evidence of base compositional heterogeneity; aside from third codon positions in fish, reptiles, and tetrapods. Third, the SWS1 data set exhibits a relatively unskewed distribution of substitution rate frequencies among the different types of substitutions, and a substantial amount of among site rate variation, both of which are characteristics that previous studies suggest might be important for phylogenetic utility [59,65,78]. Fourth, the substitution rate frequencies are not only unskewed, they are also relatively constant across the phylogeny, indicative of a homogeneous substitution process, which may be important in not attenuating phylogenetic signal across fairly large divergences.

Why does the SWS1 visual pigment gene exhibit useful phylogenetic characteristics across such a wide range of divergences in vertebrates? Factors important in contributing to its utility as a phylogenetic marker may be due, at least in part, to the highly conserved, yet somewhat variable nature of its functional role in visual transduction. The fundamental role of SWS1 genes in mediating visual sensitivities at the shortest wavelengths of the spectrum is highly conserved throughout vertebrates, along with its expression in a specific photoreceptor cell in the retina characterized by extremely short outer segments [119-121]. Unlike many other genes used for systematic purposes, which are often housekeeping genes which tend to be expressed ubiquitously in many different cell types, or developmental genes that may be expressed in a different tissues mediating a variety of functions, SWS1 genes are generally only expressed in a particular photoreceptor cell type, though they have been found in rare instances to be co-expressed in other types of photoreceptors with longer wavelength-sensitive opsin genes, for example in the mouse [122], guinea pig [7], and tiger salamander [123]. Its overall tertiary 3D structure has remained unchanged, most likely due to constraints imposed by its role as an integral membrane protein, and the mechanisms of activation which require specific structural rotations of the helices which are thought to be conserved in many G-protein coupled receptors [124]. This conserved role in evolution may be important for maintaining homogeneous molecular evolutionary processes such as substitution rate frequencies across vertebrate evolution.

Along with its highly conserved role as the first step in visual transduction, vertebrate SWS1 visual pigments are well-understood examples of functional variation in spectral sensitivity: SWS1 pigments can range in maximal absorption from the ultraviolet to violet [see [12,125] for reviews]. However, these shifts in function are thought to be mediated via a few specific residues in the protein [see [12]]. Despite the obvious constraints on protein structure, and adaptive changes in function such as spectral sensitivities, these factors may have only limited influence in restricting protein sequence evolution, as SWS1 clearly shows a substantial amount of evolutionary variation capable of carrying phylogenetic information. In fact, the constraints imposed by SWS1 structure and function may provide a framework in which the protein can vary in a more homogeneous fashion that allows for the retention of a strong phylogenetic signal.

## Conclusion

The various phylogenetic methods used to analyse SWS1 produced strongly supported topologies showing remarkable congruence with most traditionally accepted hypotheses of vertebrate evolution from the consensus of morphological and molecular studies. This nuclear, protein coding gene recovers not only deep relationships, usually requiring combinations of genes, but also recent relationships that typically require markers with high rates of evolution, such as mitochondrial DNA. The few exceptions include the monophyly of the primates, the relationships among the major groups of placental mammals, and the position of the guinea pig, which remain inconclusive in many data sets.

The phylogenetic utility of the SWS1 gene may result from a number of features of the SWS1 gene found to be important in previous studies, including substantial among site rate variation. Its ability to carry a phylogenetic signal across a broad range of divergences in vertebrates may also be due to a number of features, such as fairly homogeneous substitution rate matrix parameters, which are potentially important but largely unexplored for other phylogenetic markers. Future studies will explore these characteristics in data sets of other nuclear markers, in order to assess how well they correlate with phylogenetic utility.

## Methods

### Sequence alignment

Sixty two vertebrate SWS1 opsin nucleotide and amino acid sequences were retrieved from GenBank, with accession numbers for all sequences used in the analyses presented here provided in Table 3. SWS1 coding sequences range in length from 1005 (salmonids) to 1056 (pig) nucleotides, with very few indels (only 6 indels in complete coding sequences in the entire alignment; see Table 3 and Additional file 1). All SWS1 opsin genes identified so far have four introns at highly conserved homologous positions (located at amino acid positions 120, 176, 231, and 311 in the macaque sequence [126]). The first two introns are generally short, ranging in length from 70–76 bp in fish (*Dimidiochromis compressiceps*), to 283–324 bp in mammals (*Macaca fascicularis*); whereas the second two introns tend to be longer (120–143 bp in *D. compressiceps*, 627–979 bp in *M. fascicularis*) [126,127]. Only one copy of SWS1 has been found in all taxa investigated so far, with the exception of the smelt (*Plecoglossus altivelis*), which may be due to a unique duplication specific to this lineage of fish [128]. Only one smelt sequence was included in our analyses, as investigations including the second sequence showed it to be strongly monophyletic with the first, and had no other effect on the phylogeny (results not shown).

**Table 3:** Accession numbers and species identification for taxa in SWS1 data set

**Class (order)**	**Common Name**	**Scientific name**	**Accession #**
Actinopterygii			
*Cyprinodontiformes*	killifish	*Lucania goodei*	AY296735
*Perciformes*	Malawi eye-biter	*Dimidiochromis compressiceps*	AF191220
	Fuelleborn's cichlid	*Labeotropheus fuelleborni*	AF191223
	zebra mbuna	*Metriaclima zebra*	AF191219
	Nile tilapia	*Oreochromis niloticus*	AF191221
*Pleuronectiformes*	Atlantic halibut	*Hippoglossus hippoglossus*	AF156264
*Cypriniformes*	goldfish	*Carassius auratus*	D85863
	carp	*Cyprinus carpio*	AB113669
	zebrafish	*Danio rerio*	AB087810
*Osmeriformes*	smelt	*Plecoglossus altivelis*	AB098706
*Salmoniformes*	pink salmon	*Oncorhynchus gorbuscha*	AY214153
	chum salmon	*Oncorhynchus keta*	AY214143
	coho salmon	*Oncorhynchus kisutch*	AY214148
	rainbow trout	*Oncorhynchus mykiss*	AF425074
	sockeye salmon	*Oncorhynchus nerka*	AY214158
	chinook salmon	*Oncorhynchus tshawytscha*	AY214138
	Atlantic salmon	*Salmo salar*	AY214133
Amphibia			
*Anura*	African clawed frog	*Xenopus laevis*	BC084882
	American bullfrog	*Rana catesbeiana*	AB001983
*Caudata*	tiger salamander	*Ambystoma tigrinum*	AF038948
	firebelly newt	*Cynops pyrrhogaster*	AB052889
Aves			
*Galliformes*	chicken	*Gallus gallus*	NM_205438
*Ciconiiformes*	Humboldts penguin	*Spheniscus humboldti*	AJ277991
*Columbiformes*	pigeon	*Columba livia*	AJ238856
*Psittaciformes*	budgerigar	*Melopsittacus undulatus*	Y11787
*Passeriformes*	common canary	*Serinus canaria*	AJ277922
	Siberian rubythroat	*Luscinia calliope*	AY274226
	bluethroat	*Luscinia svecica*	AY274225
	blue tit	*Parus caeruleus*	AY274220
	great tit	*Parus major*	AY274221
	marsh tit	*Parus palustris*	AY274222
	yellow crowned bishop	*Euplectes afer*	AY274223
	red bishop	*Euplectes orix*	AY274224
	zebra finch	*Taenopygia guttata*	AF222331
Mammalia			
*Artiodactyla*	cow	*Bos taurus*	U92557
	pig	*Sus scrofa*	NM_214090
*Carnivora*	dog	*Canis familiaris*	XM_539386
*Primates*	white-tufted-ear marmoset	*Callithrix jacchus*	L76201
	mantled howler monkey	*Alouatta palliata*	AH005790
	weeping capuchin monkey	*Cebus olivaceus*	AH005810
	Bolivian squirrel monkey	*Saimiri boliviensis*	U53875
	crab-eating macaque	*Macaca fascicularis*	AF158977
	talapoin	*Miopithecus talapoin*	L76226
	gorilla	*Gorilla gorilla*	AH005811
	human	*Homo sapiens*	AH003620
	chimpanzee	*Pan troglodytes*	AH005813
	brown lemur	*Eulemur fulvus*	AB111464
	western tarsier	*Tarsius bancanus*	AB111463
*Proboscidea*	African elephant	*Loxodonta africana*	AY686753
*Rodentia*	guinea pig	*Cavia porcellus*	AY552608
	Djungarian hamster	*Phodopus sungorus*	AY029604
	house mouse	*Mus musculus*	AH005191
	Norway rat	*Rattus norvegicus*	AF051163
*Sirenia*	Caribbean manatee	*Trichechus manatus*	AY228443
*Dasyuromorpha*	dunnart	*Sminthopsis crassicaudata*	AY442173
*Peramelemorpha*	short nosed bandicoot	*Isoodon obesulus*	AY726544
*Diprotodontia*	tammar wallaby	*Macropus eugenii*	AY286017
	quokka	*Setonix brachyurus*	AY726545
Squamata			
	tokay gecko	*Gekko gecko*	AY024356
	day gecko	*Phelsuma madagascariensis*	AF074045
	green anole	*Anolis carolinensis*	AH007736
Cephalaspidomorphi			
*Petromyzontiformes*	pouched lamprey	*Geotria australis*	AY366495

Sampling within the vertebrate groups was as follows: one lamprey (*Geotria australis*), 17 actinopterygians (all of which were teleosts); four lissamphibians (referred to in the text as amphibians); 13 birds; three squamates; and 23 mammals (Table 3). The amino acid sequences were aligned using ClustalX [21], Additional file 1). This amino acid alignment was then used to produce an equivalently aligned nucleotide sequence alignment.

### Phylogenetic analyses

Phylogenetic analyses were performed using PAUP*v4b10, [129] for the maximum parsimony (MP) and likelihood (ML) methods, and MrBayes version 3.1 [130] for the Bayesian analyses. For the MP analysis all characters were assigned equal weight. Heuristic searches, with random addition of taxa and TBR branch swapping, were performed with 10000 random-addition sequences. A strict consensus tree was calculated from the equally most parsimonious trees found. To assess support for internal branches, bootstrap analyses [31] of 1000 replicates with 10 random-addition sequences for each replicate, were performed.

ModelTest [27] was used to perform a series of nested likelihood ratio tests in order to determine which nucleotide model of those tested best fit the data. This model was then used in subsequent model-based phylogenetic analyses such as likelihood and Bayesian analyses. Heuristic ML analyses were conducted with TBR branch swapping (10 random addition replicates), as well as bootstrap analyses with 100 replicates in order to assess the robustness of the clades recovered [31]. The Bayesian analyses were run for two million generations with default priors, sampling the chains every 100 generations. To ensure that our analyses were not trapped in local optima, four independent Markov Chain Monte Carlo (MCMC) runs were performed (with default heating values). Stationarity was assumed when the cumulative posterior probabilities of all clades stabilized. The first 5000 trees were considered 'burn-in' and discarded, and the remaining trees were saved. The associated Bayesian posterior probabilities were calculated from the sample points after the MCMC algorithm started to converge.

### Nucleotide composition and substitution frequencies

Parameters such as base frequencies, substitution rate frequencies, among site rate variation (α), and invariant sites (I) were all estimated on the ML phylogeny using maximum likelihood methods under the GTR+I+Γ model [28-30] as implemented in PAUP*. Chi-squared tests of base compositional homogeneity were also implemented in PAUP* [129]. Since estimates of invariant sites (*I*) can be problematic, particularly in reduced data partitions due to insufficient data [58], the number of invariant sites was therefore also calculated by simple counts of the observed number of constant sites in our data set, as implemented in MEGA3 [131].

## Abbreviations

SWS1, short-wavelength sensitive 1 opsin gene; SWS2, short-wavelength sensitive 2 opsin gene; RH1, rod opsin gene; RH2, rod opsin-like gene; GPCR, G-protein coupled receptor; MP, maximum parsimony; ML, maximum likelihood.

## Authors' contributions

IVH, JM and BC conceived of the study, assembled and refined the data set. IVH and FS carried out phylogenetic analyses. IVH carried out statistical analyses of sequence data. BC and IVH drafted the manuscript, with help from JM and FS. BC guided the analyses, and was involved in all aspects of the work. All authors were responsible for interpretation of results in an evolutionary context as well as read and approved the final version of the manuscript.

## Supplementary Material

Additional File 1 Amino acid alignment of vertebrate SWS1 opsin sequences Click here for file
