# Supplementary material for: Short-wavelength sensitive opsin (SWS1) as a new marker for vertebrate phylogenetics
Source: BMC Evol Biol. 2006 Nov 15;6:97. doi: 10.1186/1471-2148-6-97 (PMC1664589; doi:10.1186/1471-2148-6-97)
Supplement: Additional File 1 — Amino acid alignment of vertebrate SWS1 opsin sequences [file 1471-2148-6-97-S1.pdf]

```
pouched_lamprey        -----MSGDE EFYLFKNISK VGPWDGPQF- HIAPKWAFYL QAAFMGFVFI CGTPLNAIVL VVTIKYKKLR  [ 70]
goldfish               -------MDA WTYQFGNLSK ISPFEGPQY- HLAPKWAFYL QAAFMGFVFF VGTPLNAIVL FVTMKYKKLR  [ 70]
carp                   -------MDA WTYQFGNLSK VSPFEGPQY- HLAPKWAFYL QAAFMGFVFL VGTPMNAIVL FVTMKYKKLR  [ 70]
Malawi_eye-biter       --------GK HFHLYENISK ISPFEGPQY- YLAPVWAFYL QAAFMGFVFF AGTPLNFIVL VATMKYKKLR  [ 70]
zebrafish              -------MDA WAVQFGNASK VSPFEGEQY- HIAPKWAFYL QAAFMGFVFI VGTPMNGIVL FVTMKYKKLR  [ 70]
Atlantic_halibut       ------MMGK HFHLYENVSN VSPFDGPQY- YLAPQWAFHL QTIFMGSVLF AGTPLNFIVL LVTLKYKKLR  [ 70]
Fuellerborns_cichlid   -------MGK HFHLYENISK ISPFEGPQY- YLAPVWAFYL QAAFMGFVFF AGTPLNFIVL VATMKYKKLR  [ 70]
Nile_tilapia           -------MGK YFHLYENISK VSPFEGPQY- YLAPTWAFYL QAAFMGFVLF AGTPLNFVVL VATMKYKKLR  [ 70]
killifish              -------MGK HFHLYENISK VDPFEGPQY- YLAPSWAFHL QALFMGFVFF TGTPLNFLVL LATAKYKKLR  [ 70]
zebra_mbuna            -------MGK HFHLYENISK ISPFEGPQY- YLAPVWAFYL QAAFMGFVFF AGTPLNFIVL VATMKYKKLR  [ 70]
pink_salmon            -------MGK DFHLYENISK VSPFEGPQY- HLAPKWAFYL QTAFMGFVFF AGTPLNFIIL LVTVKYKKLR  [ 70]
chum_salmon            -------MGK DFHLYENISK VSPFEGPQY- HLAPIWAFYL QTAFMGFVFF AGTPLNFIIL VVTVKYKKLR  [ 70]
coho_salmon            -------MGK NFHLYENISK VSPFEGPQY- HLAPIWAFYL QTAFMGFVFF AGTPLNLIIL VVTVKYKKLR  [ 70]
rainbow_trout          -------MGK DFHLYENISK VSPFEGPQY- HLAPIWAFYL QTAFMGFVFF AGTPLNFIIL VVTVKYKKLR  [ 70]
sockeye_salmon         -------MGK DFHLYENISK VSPFEGPQY- HLAPIWAFYL QTAFMGFVFF AGTPLNFIIL VVTVKYKKLR  [ 70]
chinook_salmon         -------MGK DFHLYENISK VSPFGGPQY- HLAPIWAFYL QTAFMGFVFF AGTPLNFIIL VETVKYKKLR  [ 70]
Atlantic_salmon        -------MGK DFHLYENISK ISPFEGPQY- HLASMWAFYL QAAFMGFVFF AGTPLNFIIL VVTVKYKKLR  [ 70]
smelt                  -------MGK QFHLYENVSK IGPFEGPQY- YLAPVWVFYL QAAFMGFVFF VGTPLNFVIL MVTIKYKKLR  [ 70]
howler_monkey          --MSKMSEEE EFYLFKNISS VGPWDGPQY- HIAPVWAFYL QAAFMGIVFL AGLPLNAMVL VATVRYRKLR  [ 70]
cow                    --MSKMSEEE EFLLFKNISL VGPWDGPQY- HLAPVWAFHL QAVFMGFVFF VGTPLNATVL VATLRYRKLR  [ 70]
dog                    --MSKMSGEE EFYLFKNISL VGPWDGPQY- HIAPVWAFHL QAVFMGFVFF AGTPLNGTVL VATLRYKKLR  [ 70]
marmoset               --MSKMSEEE EFYLFKNISS VGPWDGPQYP HIAPSWAYYL QAAFMGIVFL IGLPLNTMVL VATVRYKKLR  [ 70]
capuchin_monkey        --MSKMSEEE EFYLFKNISS VGPWDGPQY- HIAPVWAFYL QAAFMGIVFL VGLPLNAMVL VATVRYKKLR  [ 70]
guinea_pig             -----MSEEE EFYLFKNASS VGPWDGPQY- HVAPVWAFRL QAAFMGIVFC IGTPLNGIVL VATLLYKKLR  [ 70]
brown_lemur            --MCKMSGEE EFYLFKNLSP VGPWDGPQY- HIAPVWAFYL QAAFMGLVFF AGAPLNVMVL VATLRYKKLR  [ 70]
gorilla                ---MRKMSEE EFYLFKNISP VGPWDGPQY- HIAPVWAFYL QAAFMGTVFL IGFPLNAMVL VATLRYKKLR  [ 70]
human                  ---MRKMSEE EFYLFKNISS VGPWDGPQY- HIAPVWAFYL QAAFMGTVFL IGFPLNAMVL VATLRYKKLR  [ 70]
bandicoot              -----MSGDE EFYLFKNISS VGPWDGPQY- HIAPAWAFHC QTVFMGFVFF AGTPLNAVVL IATLRYKKLR  [ 70]
African_elephant       -MIKMSGEEE EFYLFKNISS VGPWGGPQY- HIGPVWAFHL QAAFMAFVFF VGTPLNALVL VATLRYRKLR  [ 70]
wallaby                -----MSGDE EFYLFKNISS VGPWDGPQY- HIAPAWAFHC QTVFMGFVFF AGTPLNAVVL IATFRYKKLR  [ 70]
macaque                --MRKMSEEE EFYLFKNLSS VKPWDGPQY- HIAPVWAFYL QAAFMGTVFL AGFPLNAMVL VATVRYKKLR  [ 70]
house_mouse            -----MSGED DFYLFQNISS VGPWDGPQY- HLAPVWAFRL QAAFMGFVFF VGTPLNAIVL VATLHYKKLR  [ 70]
talapoin               --MRKMSEEE EFYLFKNISS VKPWDGPQY- HIPPVWAYYL QAAFMGTVFL AGFPLNAMVL VATVRYKKLR  [ 70]
Djungarian_hamster     ---------- ---------- ---------- ---------- -------VFF AGVPLNATVL VATLRYKKLR  [ 70]
chimpanzee             ---MRKMSEE EFYLFKNISS VGPWDGPQY- HIAPVWAFYL QAAFMGTVFL IGFPLNAMVL VATLRYKKLR  [ 70]
Norway_rat             -----MSGE? EFYLFQNISS VGPWDGPQY- HIAPVWAFHL QAAFMGFVFF AGTPLNATVL VATLHYKKLR  [ 70]
squirrel_monkey        --MSKMPEEE EFYLFKNISS VGPWDGPQY- HIAPVWAFQL QAAFMGIVFL AGLPLNSMVL VATVRYKKLR  [ 70]
quokka                 -----MSGDE EFYLFKNISS VGPWDGPQY- HIAPAWAFHC QTVFMGFVFF AGTPLN?VVL IATFRYKKLR  [ 70]
dunnart                -----MSGDE EFYLFKNISL VGPWDGPQY- HLAPAWAFHF QTAFMGFVFF AGTSLNGVVL IATLRYKKLR  [ 70]
pig                    MSKMPEEEEE EFLLFKNISL VGPWDGPQY- HLAPVWVFHL QAAFMGFVFL VGTPLNATVL VATLRYRKLR  [ 70]
western_tarsier        -----MSEEE EFYLFKNISS VGPWDGPQH- HLAPVWVFHL QAAFMGVVFS VGIPLNTMVL VATLRYKKLR  [ 70]
Caribbean_manatee      --MSKMSEEE EFYLFKNISS VGPWDGPQY- HIAPVWAFRL QAAFMGFVFF AGTPLNAMVL VATLRYRKLR  [ 70]
green_anole            -----MSGQE DFYLFENISS VGPWDGPQY- HIAPMWAFYF QTAFMGFVFF AGTPLNAIIL IVTVKYKKLR  [ 70]
tokay_gecko            -----MSGEE DFYLYANISS VGPFDGPQY- HIAPMWAFYF QTAFMGFVFF VGTPLNAIIL FAIVKYKKLR  [ 70]
day_gecko              -----MSGEE DFYLFTNISS VGPFDGPQY- HIAPMWAFYI QTAFMGFVFF AGTPLNGIIL IAIVKYKKLR  [ 70]
pigeon                 -----MSGDE EFYLFKNGSS VGPWDGPQY- HIAPPWAFYL QTAFMGFVFL VGTPFNAIVL VVTIKYKKLR  [ 70]
yellow-crowned_bishop  ---------- ---------- ---------- ------AFYL QTIFMGLVFV AGTPLNAIVL IVTIKYKKLR  [ 70]
red_bishop             ---------- ---------- ---------- ------AFYL QTIFMGLVFV AGTPLNAIVL IVTIKYKKLR  [ 70]
chicken                -----MSSDD DFYLFTNGSV PGPWDGPQY- HIAPPWAFYL QTAFMGIVFA VGTPLNAVVL WVTVRYKRLR  [ 70]
Siberian_rubythroat    ---------- ---------- ---------- -------FYL QTIFMGLVLV AGTPLNAIVL IVTVKYKKLR  [ 70]
bluethroat             ---------- ---------- ---------- ------?FYL QTIFMGLVLV AGTPLNAIVL IVTVKYKKLR  [ 70]
budgerigar             -----MSGEE EFYLFKNGSI GGPWDGPQY- HIAPPWAFYL QTAFMGFVFM VGTPLNAIVL VVTIKYKKLR  [ 70]
blue_tit               ---------- ---------- ---------- -----WAFYL QTIFMGLVFV AGTPLNAIVL IVTIKYKKLR  [ 70]
great_tit              ---------- ---------- ---------- ------AFYL QTIFMGLVFV AGTPLNAIVL IVTIKYKKLR  [ 70]
marsh_tit              ---------- ---------- ---------- ------AFYL QTIFMGLVFV AGTPLNAIVL IVTIKYKKLR  [ 70]
Humboldts_penguin      ---------- ---------- --PWDGPQY- HIAPPWAFYL QTAFMGFVFL VGTPFNAVVL VVTVKYKKLR  [ 70]
common_canary          ------MDEE EFYLFKNQSS VGPWDGPQY- HIAPMWAFYL QTIFMGLVFV AGTPLNAIVL IVTVKYKKLR  [ 70]
zebra_finch            ------MDEE EFYLFKNQSS VGPWDGPQY- HIAPMWAFYL QTIFMGLVFV AGTPLNAIVL IVTIKYKKLR  [ 70]
tiger_salamander       -----MLEEE EFYLYKNISK VGPWDGPQY- HIAPAWTFYF QTAFMGFVFF VGTPLNAIVL IVTVKYKKLR  [ 70]
firebelly_newt         -----MVGDD DFYLFKNISK VGPWDGPQY- HIAPAWTFYF QTAFMGFVFF VGTPLNAIVL IVTVKYKKLR  [ 70]
American_bullfrog      -----MLGDE DFYLFKNVSD IRPWDGPQY- HIAPRWAFTL QAIFMGVVFI IGTPLNAVVL LVTVKYKKLR  [ 70]
African_clawed_frog    -----MLEEE DFYLFKNVSN VSPFDGPQY- HIAPKWAFTL QAIFMGMVFL IGTPLNFIVL LVTIKYKKLR  [ 70]

pouched_lamprey        QPLNYILVNI SAAGLVFCLF SISTVFVASM QGYFFLGPTI CALEAFFGSL AGLVTG-WSL AFLAAERYIV  [140]
goldfish               QPLNYILVNI SLGGFIFDTF SVSQVFFSAL RGYYFFGYTL CAMEAAMGSI AGLVTG-WSL AVLAFERYVV  [140]
carp                   QPLNYILVNI SLGGFIFDTF SVSQVFFSAL RGYFFFGHTM CAMESAMGSI AGLVTG-WSL AVLAFERYVV  [140]
Malawi_eye-biter       VPLNFILVNI SFSGFIFVTF SVSQVFLASM RGYYFLGHTL CALESAVGSV AGLVTA-WSL AVLSFERYLV  [140]
zebrafish              QPLNYILVNI SLAGFIFDTF SVSQVFVCAA RGYYFLGYTL CAMEAAMGSI AGLVTG-WSL AVLAFERYVV  [140]
Atlantic_halibut       VPLNYILVNI CFAGLIFVVF SVSQVFVSTM RGYFFLGPTL CALESAMGSI AGLVTS-WSL AVLSLERYLV  [140]
Fuellerborns_cichlid   VPLNFILVNI SFSGFIFVTF SVSQVFLASM RGYYFLGHTL CALESAVGSV AGLVTA-WSL AVLSFERYLV  [140]
Nile_tilapia           VPLNFILVNI SFSGFIFVTF SVSQVFLASM RGYYFLGHTL CALEAAVGAI AGLVTA-WSL AVLSFERYLV  [140]
```

```
killifish             APLNYILVNI SFAGFIFVTF SVSQVFVSSA RGYYFLGYTL CALEAAMGAV AGLVTS-WSL AVLSFERYLV  [140]
zebra_mbuna           VPLNFILVNI SFSGFIFVTF SVSQVFLASM RGYYFLGHTL CALESAVGSV AGLVTA-WSL AVLSFERYLV  [140]
pink_salmon           QPLNYILVDV SLAGFIFVTF SVSQVFVSSA RGYYFLGYTL CAMEACMGSI AGLVSA-WSL AVLAFERYVV  [140]
chum_salmon           QPLNYILVNV SLAGFIFVTF SVSQVFVSSA RGYYFLGYTL CAMEACMGSI AGLVSA-WSL AVLAFERYVV  [140]
coho_salmon           QPLNYILVNV SLAGFIFVTF SVSQVFVSSA GGYYFLGYTL CAMEACMGSI AGLVSA-WSL AVLAFERYVV  [140]
rainbow_trout         QPLNYILVNV SLAGFIFVTF SVSQVFVSSA RGYYFLGYTL CAMEACMGSI AGLVSA-WSL AVLAFERYVV  [140]
sockeye_salmon        QPLNYILVNV SLAGFIFVTF SVSQVFVSSA RGYYFLGYTL CAMEACMGSI AGLVSA-WSL AVLAFERYLV  [140]
chinook_salmon        QPLNYILVNV SLAGFIFVTF SVSQVFVSSA RGYYFLGYTL CAMEACMGSI AGLVSA-WSL AVLAFERYVV  [140]
Atlantic_salmon       QPLNFILVNI SLAGFIFVTF SVSQVFVSSA RGYYFLGYTL CAMEACMGSI AGLVSA-WSL AVLAFERYVV  [140]
smelt                 QPLNYILVNI SVGGFIFAVF SVSQVFVASL RGYYFLGYTL CAMEACMGSI AGLVTG-WSL AVLAFERYVV  [140]
howler_monkey         HPLNYVLVNV SVGGFLLCIF SVFPVFVASC HGYFVFGRHV CALEGFLGTV AGLVTG-WSL AFLAFERYIV  [140]
cow                   QPLNYILVNV SLGGFIYCIF SVFIVFITSC YGYFVFGRHV CALEAFLGCT AGLVTG-WSL AFLAFERYII  [140]
dog                   QPLNYILVNV SLGGFLYCIS VS-TVFIASC QGYFVFGRHV CALEAFLGST AGLVTG-WSL AFLAFERYIV  [140]
marmoset              HPLNYVLVNV SVGGFLLCIF SVFPVFVASC HGYFVFGRHV CALEGFLGTV AGLVTG-WSL AFLAFERYIV  [140]
capuchin_monkey       HPLNYVLVNV SVGGFLLCIF SVFPVFVASC HGYFVFGRHV CALEAFLGTV AGLVTG-WSL AFLAFERYIV  [140]
guinea_pig            QPLNYILVNV SLGGFLVCIF SVLAVFIASC YGYFIFGRHV CALEGFLGSV AGMVTG-WSL AFLAFERYLV  [140]
brown_lemur           QPLNYILVNL SFGGFLCCIF SVLPVFIASC RGYFLFGRHV CALEGFLGSA AGLVIG-WSL AFLAFERYVI  [140]
gorilla               QPLNYILVNV SFGGFLLCIF SVFPVFVASC NGYFVFGRHV CALEGFLGTV AGLVTG-WSL AFLAFERYIV  [140]
human                 QPLNYILVNV SFGGFLLCIF SVFPVFVASC NGYFVFGRHV CALEGFLGTV AGLVTG-WSL AFLAFERYIV  [140]
bandicoot             QPLNYILVNI SLAGFIFCIF SVFTVFISSS QGYFIFGRHV CAMEAFLGSV AGLVTG-WSL AFLAFERFIV  [140]
African_elephant      QPLNYILVNI SLGGFLSCIF SVFIVFISSC KGYFIFGRYV CALEAFVGSV AGLVTG-WSL AFLAFERYIV  [140]
wallaby               QPLNYILVNI SLAGFIYCIF SVFTVFISSS QGYFIFGRHV CAMEGFLGSV AGLVTG-WSL AFLAFERFIV  [140]
macaque               QPLNYILVNV SFGGFLLCIF SVFPVFVNSC KGYFVFGRHV CAFEAFLGTV AGLVTG-WSL AFLAFERYIV  [140]
house_mouse           QPLNYILVNV SLGGFLFCIF SVFTVFIASC HGYFLFGRHV CALEAFLGSV AGLVTG-WSL AFLAFERYVV  [140]
talapoin              QPLNYILVNV SFGGFLLCIF SVFPVFVNSC KGYFVFGRHV CGFEAFLGTV AGLVTG-WSL AFLAFERYIV  [140]
Djungarian_hamster    QPLNYILVNI SLGGFLFCSF SVFTVFIASC HGYFLFGRHV CALEAFLGSV AGLVTG-WSL AFLAFERYIV  [140]
chimpanzee            QPLNYILVNV SFGGFLLCIF SVFPVFVASC NGYFVFGRHV CALEGFLGTV AGLVTG-WSL AFLAFERYIV  [140]
Norway_rat            QPLNYILVNV SLGGFLFCIF SVFTVFIASC HGYFLFGRHV CALEAFLGSV AGLVTG-WSL AFLAFERYLV  [140]
squirrel_monkey       HPLNYVLVNV SVGGFLLCIF SVLPVFVNSC NGYFVFGRHV CALEGFLGTV AGLVTG-WSL AFLAFERYIV  [140]
quokka                QPLNYILVNI SLAGFIYCII SVFTVFISSS QGYFIFGRHV CAMEGFLGSV AGLVTG-WSL AFLAFERFIV  [140]
dunnart               QPLNYILVNI SLAGFIFCVF SVFTVFVSSS QGYFVFGRHV CAMEGFLGSV AGLVTG-WSL AFLAFERFIV  [140]
pig                   QPLNYILVNV SLGGFIYCIF SVFSVFIASC HGYFVFGRRV CAMEAFLGSA AGLVTG-WSL AFLAFERYII  [140]
western_tarsier       QPLNYILVNV SLGGFLLCIF SVLPVFIASC RGYFVFGRHV CALEGFLGSV AGLVTG-WSL AFLAFERYMV  [140]
Caribbean_manatee     QPLNYILVNI SLGGFLYCIF SVFIVFITSC HGYFIFGRYV CALEAFLGAT TGLVTG-WSL AFLAFERYIV  [140]
green_anole           QPLNYILVNI SFAGFLFCTF SVFTVFMASS QGYFFFGRHV CAMEAFLGSV AGLVTG-WSL AFLAFERYIV  [140]
tokay_gecko           QPLNYILVNI SAAGFLFCVV AVFTVFISSS QGYFIFGKHI CALEAFLGSL AGLVTG-WSL AFLALERYIV  [140]
day_gecko             QPLNYILVNI SAAGFLFCTF SVFTVFVSSA QGYFVFGKHI CALEAFLGSL AGLVTG-WSL AFLAMERYIV  [140]
pigeon                QPLNYILVNI SFSGFISCIF SVFTVFVSSS QGYFIFGKDM CALEAFVGAT GGLVTG-WSL AFLAFERYIV  [140]
yellow-crowned_bishop QPLNYILVNI SVSGLMCCVF CIFTVFVASS QGYFVFGKHM CAFEGFAGAT GGLVTG-WSL AFLAFERYIV  [140]
red_bishop            QPLNYILVNI SVSGLMCCVF CIFTVFVASS QGYFVFGKHM CAFEGFAGAT GGLVTG-WSL AFLAFERYIV  [140]
chicken               QPLNYILVNI SASGFVSCVL SVFVVFVASA RGYFVFGKRV CELEAFVGTH GGLVTG-WSL AFLAFERYIV  [140]
Siberian_rubythroat   QPLNYILVNI SVSGLMCCVF CIFTVFVSSS QGYFVFGKHV CAFEGFSGAT VGLVTG-WSL AFLAFERYIV  [140]
bluethroat            QPLNYILVNI SVSGLMCCVF CIFTVFVSSS QGYFVFGKHV CAFEGFSGAT VGLVTG-WSL AFLAFERYIV  [140]
budgerigar            QPLNYILVNI SFCGFLACII CIFTVFVSSS QGYFVFGKHV CAFEGFMGAT AGLVTG-WSL AFLAFERYIV  [140]
blue_tit              QPLNYILVNI SVSGLMCCVF CIFTVFVSSS QGYFVFGKHM CAFEGFAGAT GGLVTG-WSL AFLAFERYIV  [140]
great_tit             QPLNYILVNI SVSGLMCCVF CIFTVFVSSS QGYFVFGKHM CAFEGFAGAT GGLVTG-WSL AFLAFERYIV  [140]
marsh_tit             QPLNYILVNI SVSGLMCCVF CIFTVFVSSS QGYFVFGKHM CAFEGFAGAT GGLVTG-WSL AFLAFERYIV  [140]
Humboldts_penguin     QPLNYILVNI SFSGFISCIF SVFTVFVSSS QGYFVFGKHM CALEGFVGAT GGLVTG-WSL AFLAFERYIV  [140]
common_canary         QPLNYILVNI SVSGLMCCVF CIFTVFVASS QGYFVFGKHM CRFEGFAGAT GGMVTG-WSL AFLAFERYIV  [140]
zebra_finch           QPLNYILVNI SVSGLMCCVF CIFTVFIASS QGYFVFGKHM CAFEGFAGAT GGLVTG-WSL AFLAFERYIV  [140]
tiger_salamander      QPLNYILVNV SLAGFTFCIF SVFTVFVSSS QGYFIFGKTI CELEAFLGSV SGLVTG-WSL AFLAIERYIV  [140]
firebelly_newt        QPLNYILVNV SLAGFTFCIF SVFSVFVASS QGYFIFGKFM CEMEAFLGSV SGLVTG-WSL AFLAIERYIV  [140]
American_bullfrog     QPLNYILVNI SVGGLLICIF SNFVVFINSW QGYFFFGKAF CAIEAFVGTL AGLVTG-WSL AFLAFERYIV  [140]
African_clawed_frog   QPLNYILVNI TVGGFLMCIF SIFPVFVSSS QGYFFFGRIA CSIDAFVGTL TGLVTG-WSL AFLAFERYIV  [140]

pouched_lamprey       ICKPFGNFRF GSKHALVAVG LTWMLGLSVA LPPFFGWSRY IPEGLQCSCG PDWYTVGTKY KSEYYTYFLF  [210]
goldfish              ICKPFGSFKF GQSQALGAVA LTWIIGIGCA TPPFWGWSRY IPEGIGTACG PDWYTKNEEY NTESYTYFLL  [210]
carp                  ICKPFGSFKF GQGQAMGAVA LTWVIGIGCA TPPFWGWSRY IPEGLGTSCG PDWYTKNEEY NCESYTYFLM  [210]
Malawi_eye-biter      ICKPFGAFKF GSNHALAAVA FTWFMGIGCA CPPFFGWSRY IPEGLGCSCG PDWYTHNEQY NTTSYTHFLM  [210]
zebrafish             ICKPFGSFKF GQGQAVGAVV FTWIIGTACA TPPFFGWSRY IPEGLGTACG PDWYTKSEEY NSESYTYFLL  [210]
Atlantic_halibut      ICKPFGAFRF GSNHAAGAVA FTWFMGISCA IPPFFGWSRY IPEGLGCSCG PDWYTHNEEF HCSSYTNFLM  [210]
Fuellerborns_cichlid  ICKPFGAFKF GSNHALAAVA FTWFMGIGCA CPPFFGWSRY IPEGLGCSCG PDWYTHNEQY NTTSYTHFLM  [210]
Nile_tilapia          ICKPFGAFKF GSNHALAAVA FTWFMGVGCA CPPFFGWSRY IPEGLGCSCG PDWYTHNEEY NTTSYIYFLL  [210]
killifish             ICKPFGAFKF GSSHAAGAVV FTWFMGVGCS SPPFFGWSRY IPEGLGCSCG PDWYTNDQEL GTTSYMYFLL  [210]
zebra_mbuna           ICKPFGAFKF GSNHALAAVA FTWFMGIGCA CPPFFGWSRY IPEGLGCSCG PDWYTHNEQY NTTSYTHFLM  [210]
pink_salmon           ICKPFGTFKF DNNQALAAVG FTWVMGIGCA TPPFFGWSRY IPEGLGCSCG PDWYTNNEEY HCASYTKFLI  [210]
chum_salmon           ICKPFGTFKF DNNQALAAVG FTWVMGIGCA TPPFFGWSRY IPEGLGCSCG PDWYTNNEEY HCASYTKFLI  [210]
coho_salmon           ICKPFGTFKF DNNQALAAVG FTWVMGIGCA TPPFFGWSRY IPEGLGCSCG PDWYTNNEEY HCASYTKFLI  [210]
rainbow_trout         ICKPFGTFKF DNNQALAAVG FTWVMGIGCA TPPFFGWSRY IPEGLGCSCG PDWYTNNEEY HCASYTKFLI  [210]
sockeye_salmon        ICKPFGTFKF DNNQALAAVG FTWVMGIGCA TPPFFGWSRY IPEGLGCSCG PDRYTNNEEY HCASYTKFLI  [210]
chinook_salmon        ICKPFGTFKF DNNQALAAVG FTWVMGIGCA TPPFFGWSRY IPEGLGCSCG PDWYTNNEEY HCASYTKFLI  [210]
```

```
Atlantic_salmon       ICKPFGTFKF DNNQALAAVA FTWVMGIGCA TPPFFGWSRY IPEGLGCSCG PDWYTNNEEY HCASYTKFLI  [210]
smelt                 ICKPFGQFKF GNTQALMAVG FTWVMGVGCA TPPFFGWSRY IPEGLGCACG PDWYTHNEEY HCTSYTYFLM  [210]
howler_monkey         ICKPFGNFRF SSKHALMVVL TTWTIGIGVS IPPFFGWSRF IAEGLQCSCG PDWYTVGTKY RSEYYTWFLF  [210]
cow                   ICKPFGNFRF SSKHALMVVV ATWTIGIGVS IPPFFGWSRF VPEGLQCSCG PDWYTVGTKY YSEYYTWFLF  [210]
dog                   ICKPFGNFRF SSKHALMVVL ATWTIGIGVS IPPFFGWSRF IPEGLQCSCG PDWYTVGTKY RSEYYTWFLF  [210]
marmoset              ICKPFGNFRF SSKHALMVVL TTWTIGIGVS IPPFFGWSRY IAEGLQCSCG PDWYTVGTKY RSEYYTWFLF  [210]
capuchin_monkey       ICKPFGNFRF SSKHALMVVL TTWTIGIGVS IPPFFGWSRF IAEGLQCSCG PDWYTVGTKY RSEYYTWFLF  [210]
guinea_pig            ICKPFGNFRF SSKHALIVVL ATWVIGIGVS IPPFFGWSRY MPEGLQCSCG PDWYTVGTKY RSEYFAWFLF  [210]
brown_lemur           ICKPFGNFRF SSKHALMVVL ATWTIGVGVS IPPFFGWS-F IPEGLQCSCG PDWYTVGTKY RSEYYTWFLF  [210]
gorilla               ICKPFGNFRF SSKHALTVVL ATWTIGIGVS IPPFFGWSRF IPEGLQCSCG PDWYTVGTKY RSESYTWFLF  [210]
human                 ICKPFGNFRF SSKHALTVVL ATWTIGIGVS IPPFFGWSRF IPEGLQCSCG PDWYTVGTKY RSESYTWFLF  [210]
bandicoot             ICKPFGNFRF HSKHAMMVVL ATWVIGIGVS IPPFFGWSRF IPEGLQCSCG PDWYTVGTKY RSEYYTWFLF  [210]
African_elephant      ICKPFGNIRF SSKHALMVVL ATWTIGIGVS IPPFFGWSRF LPEGLQCSCG PDWYTVGTKY RSEYYTWFLF  [210]
wallaby               ICKPFGNFRF NSKHSMMVVL ATWVIGIGVS IPPFFGWSRY IPEGLQCSCG PDWYTVGTKY RSEYYTWFLF  [210]
macaque               ICKPFGNFRF SSKHALTVVL ATWTIGIGVS IPPFFGWSRF IPEGLQCSCG PDWYTVGTKY RSESYTWFLF  [210]
house_mouse           ICKPFGSIRF NSKHALMVVL ATWIIGIGVS IPPFFGWSRF IPEGLQCSCG PDWYTVGTKY RSEYYTWFLF  [210]
talapoin              ICKPFGNFRF SSKHALTVVL ATWIIGIGVS IPPFFGWSRF IPEGLQCSCG PDWYTVGTKY RSESYTWFLF  [210]
Djungarian_hamster    ICKPFGNVRF SSKHALIVVL VTWVIGVGVS IPPFFGWSRF IPEGLQCSCG PDWYTVGTKY RSESYTWFLF  [210]
chimpanzee            ICKPFGNFRF SSKHALTVVL ATWTIGIGVS IPPFFGWSRF IPEGLQCSCG PDWYTVGTKY RSESYTWFLF  [210]
Norway_rat            ICKPFGNIRF NSKHALTVVL ITWTIGIGVS IPPFFGWSRF IPEGLQCSCG PDWYTVGTKY RSEHYTWFLF  [210]
squirrel_monkey       ICKPFGNFRF SSKHALMVVL TTWTIGIGVS IPPFFGWSRY IAEGLQCSCG PDWYTVGTKY RSEYYTWFLF  [210]
quokka                ICKPFGNFRF NSKHSMMVVL ATWVIGIGVS IPPFFGWSRY IPEGLQCSCG PDWYTVGTKY HSEYYTWFLF  [210]
dunnart               ICKPFGNFRF NSKHAMMVVL ATWIIGIGVS IPPFFGWSRY IPEGLQCSCG PDWYTVGTKY RSEYYTWFLF  [210]
pig                   ICKPFGNFRF SSKHALIAVL ATWAIGIGVS IPPFFGWSRF LPEGLQCSCG PDWYTVGTKY YSEYYTWFLF  [210]
western_tarsier       ICKPFGNFRF SPKHALMVVL ATWTIGIGVS VPPFFGWSRF IPEGLQCSCG PDWYTVDTKY HSEYYTWFLF  [210]
Caribbean_manatee     ICKPFGNIRF SSKHALMVVL ATWAIGIGVS IPPFFGWSRF MPEGLQCSCG PDWYTVGTKY HSEHYTWFLF  [210]
green_anole           ICKPFGNFRF NSKHALLVVA ATWFIGIGVS IPPFFGWSRY IPEGLQCSCG PDWYTVGTKY KSEYYTWFLF  [210]
tokay_gecko           ICKPFGNFRF SAKHASLVVA ATWFIGIGVS IPPYFGWSRF IPEGLQCSCG PDWYTVGTKY YSEYYTWFLF  [210]
day_gecko             ICKPFGNFRF NAKHASLVVA ATWVIGIGVS VPPFFGWSRY IPEGLGCSCG PDWYTVGTKY RSEYYTWFLF  [210]
pigeon                ICKPFGNFRF NSKHALMAVV ATWVIGLGVA LPPWFGWSRY VPEGLQCSCG PDWYTVGTKY KSEYYTWFLF  [210]
yellow-crowned_bishop ICKPFGNFRF NSRHALLVVA ATWIIGVGVA IPPFFGWSRY IPEGLQCSCG PDWYTVGTKY KSEYYTWFLF  [210]
red_bishop            ICKPFGNFRF NSRHALLVVA ATWIIGVGVA IPPFFGWSRY IPEGLQCSCG PDWYTVGTKY KSEYYTWFLF  [210]
chicken               ICKPFGNFRF SSRHALLVVV ATWLIGVGVG LPPFFGWSRY MPEGLQCSCG PDWYTVGTKY RSEYYTWFLF  [210]
Siberian_rubythroat   ICKPFGNFRF NSRHALLVVA ATWIIGVGVA IPPFFGWSRY IPEGLQCSCG PDWYTVGTKY KSEYYTWFLF  [210]
bluethroat            ICKPFGNFRF NSRHALLVVA ATWIIGVGVA IPPFFGWSRY IPEGLQCSCG PDWYTVGTKY KSEYYTWFLF  [210]
budgerigar            ICKPLGNFRF TAKHALVVVV ATWVIGIGVA IPPFFGWSRY VPEGLQCSCG PDWYTVGTKY RSEYYTWFLF  [210]
blue_tit              ICKPFGNFRF NSRHALLVVA ATWIIGVGVA VPPFFGWSRY VPEGLQCSCG PDWYTVGTKY KSEYYTWFLF  [210]
great_tit             ICKPFGNFRF NSRHALLVVA ATWIIGVGVA VPPFFGWSRY VPEGLQCSCG PDWYTVGTKY KSEYYTWFLF  [210]
marsh_tit             ICKPFGNFRF NSRHALLVVA ATWIIGVGVA VPPFFGWSRY VPEGLQCSCG PDWYTVGTKY KSEYYTWFLF  [210]
Humboldts_penguin     ICKPFGNFRF SSKHAVMVVV ATWVIGVGVA IPPFFGWSRY IPEGLQCSCG PDWYTVGTKY KSEYYTWLLF  [210]
common_canary         ICKPFGNFRF NSRHALLVVA ATWIIGVGVA IPPFFGWSRY IPEGLQCSCG PDWYTVGTKY KSEYYTWFLF  [210]
zebra_finch           ICKPFGNFRF NSRHALLVVA ATWIIGVGVA IPPFFGWSRY IPEGLQCSCG PDWYTVGTKY KSEYYTWFLF  [210]
tiger_salamander      ICKPMGSFRF SSKHATMVVL ATWAIGFSVS IGPLVGWSRY IPEGLQCSCG PDWYTVGTKY NSETYTWFLF  [210]
firebelly_newt        ICKPMGNFRF ASKHALMIVL TTWVIGFSVS IGPLVGWSRY IPEGLQCSCG PDWYTVGTKY NSETYTWFLF  [210]
American_bullfrog     ICKPMGTFTF TSKHALAVVL TTWMIGVGVS VPPFFGWSRY IPEGLQCSCG PDWYTVGTKY HSEYYTWFIF  [210]
African_clawed_frog   ICKPMGNFNF SSSHALAVVI CTWIIGIVVS VPPFLGWSRY MPEGLQCSCG PDWYTVGTKY RSEYYTWFIF  [210]

pouched_lamprey       VFCFVVPLSI IIFSYGSLLG TLRA--VAAQ QQESASTQKA EREVSRMVIM MVASFCTCYV PYAALAVYMV  [280]
goldfish              VSCFMMPIMI ITFSYSQLLG ALRA--VAAQ QAESASTQKA EKEVSRMVVV MVGSFVVCYG PYAITALYFS  [280]
carp                  VTCFILPMMI IIFSYSQLLG ALRA--VAAQ QAESASTQKA EKEVSRMVVV MVGSYIVCYG PYAIAALYFG  [280]
Malawi_eye-biter      VTCFIIPLSI IIFCYSQLLG ALRA--VAAQ QAESASTQKA EKEVSRMIIV MVGSFVTCYG PYALAALYFA  [280]
zebrafish             ITCFMMPMTI IIFSYSQLLG ALRA--VAAQ QAESESTQKA EREVSRMVVV MVGSFVLCYA PYAVTAMYFA  [280]
Atlantic_halibut      VTCFILPLTI IIFSYTQLLS SLRA--VAAQ QTESVSTQKA EKEVSRMIIV MVGSFVTCYG PYALAALYFA  [280]
Fuellerborns_cichlid  VTCFIIPLSI IIFCYSQLLG ALRA--VAAQ QAESASTQKA EKEVSRMIIV MVGSFVTCYG PYALAALYFA  [280]
Nile_tilapia          ITCFIFPLTI IIFCYSQLLG ALRA--VAAQ QAESASTQKA EKEVSRMIIV MVGSFVTCYG PYALAALYFA  [280]
killifish             ITCFCMPLSI IIFSYSQLLG ALRA--VAAQ QAESASTQKA EKEVSRMIVV MVGSFVTCYA PYALTGLWFA  [280]
zebra_mbuna           VTCFIIPLSI IIFCYSQLLG ALRA--VAAQ QAESASTQKA EKEVSRMIIV MVGSFVTCYG PYALAALYFA  [280]
pink_salmon           VTCFLMPMSI IFFSYSQLLG ALRA--VAAA QAESASTQKA EKEVSRMVIV MVCSFILCYG PYALAGLYFA  [280]
chum_salmon           VTCFLMPMSI IFFSYSQLLG ALRA--VAAA QAESASTQKA EKEVSRMVIV MVCSFILCYG PYALAGLYFA  [280]
coho_salmon           VTCFLMPMSI IFFSYSQLLG ALRA--VAAA QAESASTQKA EKEVSRMVIV MVCSFILCYG PYALAGLYFA  [280]
rainbow_trout         VTCFLMPMSI IFFSYSQLLG ALRA--VAAA QAESASTQKA EKEVSRMVIV MVCSFILCYG PYALAGLYFA  [280]
sockeye_salmon        VTCFLMPMSI IFFSYSQLLG ALRA--VAAA QAESASTQKA EKEVSRMVIV MVCSFILCYG PYALAGLYFA  [280]
chinook_salmon        VTCFLMPMSI IFFSYSQLLG ALRA--VAAA QAESASTQKA EKEVSRMVIV MVCSFILCYG PYALAGLYFA  [280]
Atlantic_salmon       VTCFLMPMSI IFFSYSQLLG ALRA--VAAA QAESASTQKA EKEVSRMVIV MVCSFILCYG PYALAGLYFA  [280]
smelt                 VTCFMMPLTI IIFSYSQLLG ALRA--VAAQ QAESASTQKA EKEVSRMVIV MVGSFVVCYA PYAITALYYG  [280]
howler_monkey         IFCFIVPLSL ICFSYAQLLR ALKA--VAAQ QQESATTQKA EREVSRMVVV MVGSFCVCYV PYAALAMYMV  [280]
cow                   IFCYIVPLSL ICFSYSQLLG ALRA--VAAQ QQESASTQKA EREVSHMVVV MVGSFCLCYT PYAALAMYIV  [280]
dog                   IFCFIVPLSL ICFSYLQLLR ALRA--VAAQ QQESASTQKA EREVSRMVVV MVGSFCLCYT PYAAMAMYMV  [280]
marmoset              IFCFIVPLAL ICFSYAQLLR ALKA--VAAQ QQESATTQKA EREVSRMVVV MVGSFCVCYV PYAALAMYMV  [280]
capuchin_monkey       IFCFIVPLSL ICFSYAQLLR ALKA--VAAH QQESATTQKA EREVSRMVVV MVGSFCVCYV PYAALAMYMV  [280]
guinea_pig            IFCFIVPLSL ICFSYCQLLR TLRT--VAAQ QQESATTQKA EREVSRMVVV MVGSFCVCYV PYAALAMYIV  [280]
```

```
brown_lemur           IFCFIVPLSL ICFSYSQLLR ALRA--VAAQ QQESATTQKA EREVSRMVVV MVGSFCLCYV PYAALAMYIV  [280]
gorilla               IFCFIVPLSL ICFSYTQLLR ALKA--VAAQ QQESATTQKA EREVSRMVVV MVGSFCVCYV PYAAFAMYMV  [280]
human                 IFCFIVPLSL ICFSYTQLLR ALKA--VAAQ QQESATTQKA EREVSRMVVV MVGSFCVCYV PYAAFAMYMV  [280]
bandicoot             IFCFIIPLSL ICFSYSQLLR ALRT--VAAQ QQESATTQKA EREVSRMVVV MVGSFCLCYV PYAALAMYMV  [280]
African_elephant      IFCFIVPLSL ICFSYSQLLG ALRA--VAAQ QQESATTQKA EREVSRMVVV MVASFCLCYV PYAALAMYMV  [280]
wallaby               ILCFIMPLSL ICFSYSQLLG ALRA--VAAQ QQESATTQKA EREVSRMVVM MVGSFCLCYV PYAALAMYMV  [280]
macaque               IFCFIVPLSL ICFSYTQLLR ALKA--VAAQ QQESATTQKA EREVSRMVVV MVGSFCVCYV PYAAFAMYMV  [280]
house_mouse           IFCFIIPLSL ICFSYSQLLR TLRA--VAAQ QQESATTQKA EREVSHMVVV MVGSFCLCYV PYAALAMYMV  [280]
talapoin              IFCFIVPLSL ICFSYTQLLR ALKA--VAAQ QQESATTQKA EREVSRMVVV MVGSFCVCYV PYAAFAMYMV  [280]
Djungarian_hamster    LFCFIVPLSL ICFSYSQLLR TLRA--VAAQ QQESATTQKA EREVTLMVVV MVASFCVCYV PYAALAMYMV  [280]
chimpanzee            IFCFIVPLSL ICFSYTQLLR ALKA--VAAQ QQESATTQKA EREVSRMVVV MVGSFCVCYV PYAAFAMYMV  [280]
Norway_rat            IFCFIIPLSL ICFSYFQLLR TLRA--VAAQ QQESATTQKA EREVSHMVVV MVGSFCLCYV PYAALAMYMV  [280]
squirrel_monkey       IFCFIVPLSL ICFSYAQLLR ALKA--VAAQ QQESATTQKA EREVSRMVVV MVGSFCVCYV PYAALAMYMV  [280]
quokka                ILCFIMPLSL ICFSYSQLLG ALRA--VAAQ QQESATTQKA EREVSRMVVM MVGSFCLCYV PYAALAMYMV  [280]
dunnart               IFCFIVPLSL ICFSYSQLLG ALRA--VAAQ QQESATTQKA EREVSRMVVV MVGSFCLCYV PYAAMAMYMV  [280]
pig                   IFCYIVPLAL ICFSYSQLLR ALRA--VAAQ QQESASTQKA EREVSHMVVV MVGSFCVCYT PYAALAMYIV  [280]
western_tarsier       IFCFIVPLSL ICFSYAQLLR ALRA--VAAQ QQESATTQKA EREVSRMVVV MVGSFCLCYV PYAALAMYMV  [280]
Caribbean_manatee     IFCFIVPLSL ICLLLLPAAG ALRA--VAAQ QQESATTQKA EREVSRMVIV MVGSFCLCYV PYAALAMYIV  [280]
green_anole           IFCFIVPLTL IIFSYSQLLG ALRA--VAAQ QQESATTQKA EREVSRMVVV MVGSFCLCYV PYASLAMYMV  [280]
tokay_gecko           VLCFIVPLSI IVFSYSQLLS ALRA--VAAQ QQESATTQKA EREVSRMVVV MVGSFCLCYV PYAALAMYMV  [280]
day_gecko             IFCFMVPLTI IIFSYSQLLS ALRA--VAAQ QQESATTQKA EREVSRMVVV MVGSFCTCYV PYAALAMYMV  [280]
pigeon                IFCFIVPLSL IIFSYSQLLS ALRA--VAAQ QQESATTQKA EREVSRMVVV MVGSFCLCYV PYAALAMYMV  [280]
yellow-crowned_bishop IFCFIVPLSL IIFSYSQLLS ALRA--VAAQ QQESATTQKA EREVSRMVVV MVGSFCMCYV PYAALAMYMV  [280]
red_bishop            IFCFIVPLSL IIFSYSQLLS ALRA--VAAQ QQESATTQKA EREVSRMVVV MVGSLCMCYV PYAALAMY?-  [280]
chicken               IFCFIVPLSL IIFSYSQLLS ALRA--VAAQ QQESATTQKA EREVSRMVVV MVGSFCLCYV PYAALAMYMV  [280]
Siberian_rubythroat   IFCFIVPLSL IIFSYSQLLS ALRA--VAAQ QQESATTQKA EREVSRMVVV MVGSFCLCYV PYAALAMYMV  [280]
bluethroat            IFCFIVPLSL IIFSYSQLLS ALRA--VAAQ QQESATTQKA EREVSRMVVV MVGSLCLCYV PYAALAMYMV  [280]
budgerigar            IFCFIVPLSL IIFSYSQLLS ALRA--VAAQ QQESATTQKA EREVSRMVVV MVGSFCVCYV PYAALAMYMV  [280]
blue_tit              IFCFIVPLSL IIFSYSQLLS ALRA--VAAQ QQESATTQKA EREVSRTVVV MVGSFCLCYV PYAALAMYMV  [280]
great_tit             IFCFIVPLSL IIFSYSQLLS ALRA--VAAQ QQESATTQKA EREVSRMVVV MVGSFCLCYV PYAALAMYMV  [280]
marsh_tit             IFCFIVPLSL IIFSYSQLLS ALRA--VAAQ QQESATTQKA EREVSRMVVV MVGSFCLCYV PYAGLAMYMV  [280]
Humboldts_penguin     IFCFIVPLSL IIFSYSQLLS ALRA--VAAQ QQESATTQKA EREVSRMVVV MVGSFCLCYV PYAALAMYMV  [280]
common_canary         IFCFIIPLSL IIFSYSQLLS PCGA--VAAQ QQESATTQKA EREVSRMVVV MVGSFCMCYV PYAALAMYMV  [280]
zebra_finch           IFCFIVPLSL IIFSYSQLLS ALRA--VAAQ QQESATTQKA EREVSRMVVV MVGSFCMCYV PYAALAMYMV  [280]
tiger_salamander      IFCFIIPLSL ICFCYAQLLG ALRA--VAAQ QQESATTQKA EREVTRMVIV MVVSFCLCYV PYAAMAMYMV  [280]
firebelly_newt        IFCFIIPLSL ICFCYSQLLG ALRA--VAAQ QQESATTQKA EREVTRMVIV MVASFCVCYV PYAAMAMYMV  [280]
American_bullfrog     VFCFIIPLTL ICYSYARLLG ALRA--VAAQ QQESASTQKA EKEVSRMVVV MVGSFCLCYV PYAAMAMYMI  [280]
African_clawed_frog   IFCFVIPLSL ICFSYGRLLG ALRA--VAAQ QQESASTQKA EREVSRMVIF MVGSFCLCYV PYAAMAMYMV  [280]

pouched_lamprey       TNRDHNIDLR FVTVPAFFSK ASCVYNPLIY SFMNKQFRAC ILETVCGKPI TDESETSSSR TEVSSVSTTQ  [350]
goldfish              YAEDSNKDYR LVAIPSLFSK SSCVYNPLIY AFMNKQFNAC IMETVFGKKI DESSEVSSKT ETSSVSA*--  [350]
carp                  YAEDTNKDYR LVAIPALFSK SSCVYNPLIY AFMNKQFNAC IMETVFGKKI DEGSEVSSKT ETSSVSA*--  [350]
Malawi_eye-biter      YSTDENKDYR LVTIPAFFSK SACVYNPLIY VFMNKQFNGC IMEMVFGKTM DESSEVSTKT EVSTAS*---  [350]
zebrafish             NSDEPNKDYR LVAIPAFFSK SSCVYNPLIY AFMNKQFNAC IMETVFGKKI DESSEVSSKT ETSSVSA*--  [350]
Atlantic_halibut      HSSDTNKDYR LVTIPAFFSK SSCVYNPLIY VFMNKQFKAC IMETVFGKKM DESSEVSSKT EASSVSTVN*  [350]
Fuellerborns_cichlid  YSTDENKDYR LVTIPAFFSK SACVYNPLIY VFMNKQFNGC IMEMVFGKTM DESSEVSTKT EVSTAS*---  [350]
Nile_tilapia          YSTDENKDYR LVTIPAFFSK SSCVYNPLIY VFMNKQFNGC IMEMVFGKKM DESSEVSTKT EVSTAS*---  [350]
killifish             NSPEVNKDYR LVTIPAFFSK SSCVYNPLIY AFMNKQFNAC IMEMVFGKKM EEASEVSSKT EVSTAS*---  [350]
zebra_mbuna           YSTDENKDYR LVTIPAFFSK SACVYNPLIY VFMNKQFNGC IMEMVFGKTM DESSEVSTKT EVSTAS*---  [350]
pink_salmon           YTTSENKDYR LVTIPAFFSK SSCVYNPLIY AFMNKQFNAC IMETVFGKQI EETSVSACKT EVSTA*----  [350]
chum_salmon           YTTSENKDYR LVTIPAFFSK SSCVYNPLIY AFMNKQFNAC IMETVFGKQI EETSVSASKT EVSTA*----  [350]
coho_salmon           YTTSENKDYR LVTIPAFFSK SSCVYNPLIY AFMNKQFNAC IMETVFGKQI EETSVSASKT EVSTA*----  [350]
rainbow_trout         YTTSENKDYR LVTIPAFFPK SSCVYNPLIY AFMNKQFNAC IMETVLGKQI EETSVSASKT EVSTA*----  [350]
sockeye_salmon        YTTSENKDYR LVTIPAFFSK SSCVYNPLIY AFMNKQFNAC IMETVFGKQI EETSVSASKT EVSTA*----  [350]
chinook_salmon        YTTSEVKDYR LVTIPAFFSK SSCVYNPLIY AFMNKQFNAC IMETVFGKQI EETSVSASKT EVSTA*----  [350]
Atlantic_salmon       YTTSENKDYR LVTIPAFFSK SSCVYNPLIY AFMNKQFNAC IMETVFGKQI EETSVSASKT EVSTA*----  [350]
smelt                 FSTNENKDYR MVTIPAMFSK SSCVYNPLIY AFMNKQFNAC IMETVFGKKI DESSEVSSKT ETSSVSTA*-  [350]
howler_monkey         NNRNHGLDLR LVTIPAFFSK SSCIYNPIIY CFMNKQFRAC IMEMVCGKAM TDESDLSSSQ KTEVSTVSSS  [350]
cow                   NNRNHGVDLR LVTIPAFFSK SACVYNPIIY CFMNKQFRAC IMEMVCGKPM TDESELSSSQ KTEVSTVSSS  [350]
dog                   NNRNHGLDLR LVTIPAFFSK SACVYNPIIY CFMNKQFRAC IMEMVCGKSM TEDSEMSSSQ KTEVSTVSPS  [350]
marmoset              NNRNHGLDLR LVTIPAFFSK SSCIYNPIIY CFMNKQFRAC IMEMVCGKAM TDESDISSSQ KTEVSTVS?-  [350]
capuchin_monkey       NNRNHGLDLR LVTIPSFFSK SSCIYNPIIY SFMNKQFRAC IMEMVCGKAM TDESDISSSQ KTEVSTVSTS  [350]
guinea_pig            NNRNHGLDLR LVTIPAFFSK SSCIYNPIIY CFMNKQFRAC IMELVCRKPM ADESDMSTSQ KTEVSAVSSS  [350]
brown_lemur           NNRNHGLDLR LVTIPAFFSK SACVYNPIIY CFMNKQFQAC IMEMVCGKAM TDESNTSSSQ KTEVSTFSSS  [350]
gorilla               NNRNHGLDLR LVTIPSFFSK SACIYNPIIY CFMNKQFQAC IMKMVCGKAM TDESDTCSSQ KTEVSTVSST  [350]
human                 NNRNHGLDLR LVTIPSFFSK SACIYNPIIY CFMNKQFQAC IMKMVCGKAM TDESDTCSSQ KTEVSTVSST  [350]
bandicoot             NNRNHGLDLR LVTIPAFFSK SACVYNPIIY CFMNKQFHAC IMEMICRKPM TDDSETSSSQ KTEVSTVSSS  [350]
African_elephant      NNRNHGLDLR LVTIPAFFSK SACVYNPIIY CFMNKQFRGC IMEMVCGKSV ADESDMSSSQ KMEVSTVSSS  [350]
wallaby               NNRNHGIDLR LVTIPAFFSK SSCVYNPIIY CFMNKQFHAC IMEMVCRKPM TDDSEASSSQ KTEVSTVSSS  [350]
macaque               NNRNHGLDLR LVTIPAFFSK SACIYNPIIY CFMNKQFQAH IMKMVCGKAM TDESDISSSQ KTEVSTVSSS  [350]
house_mouse           NNRNHGLDLR LVTIPAFFSK SSCVYNPIIY CFMNKQFRAC ILEMVCRKPM ADESDVSGSQ KTEVSTVSSS  [350]
```

```
talapoin              NNRNHGLDLR LVTIPAFFSK SACIYNPIIY CFMNKQFQAH IMKMVCGKAM TDESDISSSQ KTEVSTVS?-  [350]
Djungarian_hamster    NNRNHGLDLR LVTIPAFFSK SSCVYNPIIY CY?------- ---------- ---------- ----------  [350]
chimpanzee            NNRNHGLDLR LVTIPSFFSK SACIYNPIIY CFMNKQFQAC IMKMVCGKAM TDESDTCSSQ KTEVSTVSST  [350]
Norway_rat            NNRNHGLYLR LVTIPAFFSK SSCVYNPIIY CFMNKQFRAC ILEMVCRKPM TDESDMSGSQ KTEVSTVSSS  [350]
squirrel_monkey       NNRNHGLDLR LVSIPAFFSK SSCIYNPIIY CFMNKQFRAC IMEMVCGKAM TDESDISSSQ KTEVSTVSSS  [350]
quokka                NNRNHGIDLR LVTIPAFFSK SACVYNPIIY CFMNKQFHAC IMEMVCRKPM TDDSEASSSQ KTEVSTVSSS  [350]
dunnart               NNRNHGLDLR LVTIPAFFSK SACVYNPIIY CFMNKQFHAC IMEMICKKPM TDDSETTSSQ KTEVSTVSSS  [350]
pig                   NNRNHGVTLR LVTIPAFFSK SACIYNPIIY CFMNKQFRAC IMEMVCGKPM TDESDMSSSQ KTEVSTVSST  [350]
western_tarsier       NNQNHGLDLR LVTIPSFFSK SACVYNPIIY CFMNKQFQAC IMEMVCRKAM ADESDTSSSQ KTEVSALSSS  [350]
Caribbean_manatee     NNRNHGLDLR LVTIPAFFSK SACAYNPIIY CFMNKQV--- ---------- ---------- ----------  [350]
green_anole           NNRDHGLDLR LVTIPAFFSK SSCVYNPIIY CFMNKQFRAC ILETVCGKPM SDESDVSSSA QKTEVSSVSS  [350]
tokay_gecko           NNRNHGIDLR MVTIPAFFSK SSCVYNPIIY CFMNKQFRGC ILEMVCGKTM AEESEVSSAS QKTEVSSVSS  [350]
day_gecko             NYRNHGIDLR MVTIPAFFSK SACVYNPIIY CFMNKQFRGC IMEMVCGKPM SDDS-EASTS QKTEVSSVSS  [350]
pigeon                NNRNHGLDLR LVTIPAFFSK SSCVYNPIIY CFMNKQFRAC ILELVCGRPM TDDSDVSSSA QRTEVSSVSS  [350]
yellow-crowned_bishop NNREHGIDLR LVTIPAFFSK SSC?------ ---------- ---------- ---------- ----------  [350]
red_bishop            ---------- ---------- ---------- ---------- ---------- ---------- ----------  [350]
chicken               NNRDHGLDLR LVTIPAFFSK SACVYNPIIY CFMNKQFRAC IMETVCGKPL TDDSDASTSA QRTEVSSVSS  [350]
Siberian_rubythroat   NNREHGIDLR LVTIPAFFS? ---------- ---------- ---------- ---------- ----------  [350]
bluethroat            NNREHGIDLR LVTIPAFFSK SSCVYNPIIY CF?------- ---------- ---------- ----------  [350]
budgerigar            NNREHGIDLR LVTIPAFFSK SSCVYNPIIY CFMNKQFRGC IMEMVCGKPM TDDSDMSSSA QRTEVSSVSS  [350]
blue_tit              NNREHGIDLR LVTVPAFFSK SSCVYNPIIY CFMNKQFRAC IMETVCGRPM SDDSDVSSSA QRTEVSSVSS  [350]
great_tit             YNR------- ---------- ---------- ---------- ---------- ---------- ----------  [350]
marsh_tit             NNREHGIDLR LVTVPAFFSR APAFHPIIY? ---------- ---------- ---------- ----------  [350]
Humboldts_penguin     NNRNHGLDLR LVTIPAFFSK SACIYNPIIY CFMNKQFRAC IMETVCGKPM TDDSDVSSSA QRTEVSSVST  [350]
common_canary         NNREHGIDLR LVTIPAFFSK SSCVYNPIIY CFMNKQFRAC IMETVCGRPM SDDSDVSSSA QRTEVSSVSS  [350]
zebra_finch           NNREHGIDLR LVTIPAFFSK SSCVYNPIIY CFMNKQFRAC IMETVCGRPM TDDSEVSSSA QRTEVSSVSS  [350]
tiger_salamander      NNRNHGLDLR LVTIPAFFSK SACVYNPIIY SFMNKQFRAC IMETVCGTPM TDESDISSSS NKTEVSSVSS  [350]
firebelly_newt        NNRNHGLDLR LVTIPAFFSK SACVYNPIIY SFMNKQFRAC IMETVCGTPI TDESDVSTSS NKTEVSSVSS  [350]
American_bullfrog     TNRNHGLDLR FVTIPAFFSK SACVYNPIIY TFMNKQFRGC IMETVCGRPM TDDSTLSSTS QKTEVSTVSH  [350]
African_clawed_frog   TNRNHGLDLR LVTIPAFFSK SSCVYNPIIY SFMNKQFRGC IMETVCGRPM SDDSSVSSTS QRTEVSTVSS  [350]

pouched_lamprey       MIPG*----- - [361]
goldfish              ---------- - [361]
carp                  ---------- - [361]
Malawi_eye-biter      ---------- - [361]
zebrafish             ---------- - [361]
Atlantic_halibut      ---------- - [361]
Fuellerborns_cichlid  ---------- - [361]
Nile_tilapia          ---------- - [361]
killifish             ---------- - [361]
zebra_mbuna           ---------- - [361]
pink_salmon           ---------- - [361]
chum_salmon           ---------- - [361]
coho_salmon           ---------- - [361]
rainbow_trout         ---------- - [361]
sockeye_salmon        ---------- - [361]
chinook_salmon        ---------- - [361]
Atlantic_salmon       ---------- - [361]
smelt                 ---------- - [361]
howler_monkey         QVGPN----- - [361]
cow                   QVGPN*---- - [361]
dog                   QVGPN*---- - [361]
marmoset              ---------- - [361]
capuchin_monkey       QVGPN*---- - [361]
guinea_pig            KVGPH*---- - [361]
brown_lemur           QVGPN*---- - [361]
gorilla               QVGPN----- - [361]
human                 QVGPN*---- - [361]
bandicoot             QVSPS*---- - [361]
African_elephant      QVGPN*---- - [361]
wallaby               QVGPS*---- - [361]
macaque               QVGPN*---- - [361]
house_mouse           KVGPH*---- - [361]
talapoin              ---------- - [361]
Djungarian_hamster    ---------- - [361]
chimpanzee            QVGPN*---- - [361]
Norway_rat            KVGPH*---- - [361]
squirrel_monkey       QVGPN*---- - [361]
quokka                QVGPS*---- - [361]
dunnart               QVGPS*---- - [361]
pig                   QVGPN*---- - [361]
```

```
western_tarsier       QVSPN*---- - [361]
Caribbean_manatee     ---------- - [361]
green_anole           SQVSPS*--- - [361]
tokay_gecko           SQVGPS*--- - [361]
day_gecko             SQVSPS*--- - [361]
pigeon                SQVSPS*--- - [361]
yellow-crowned_bishop ---------- - [361]
red_bishop            ---------- - [361]
chicken               SQVGPT*--- - [361]
Siberian_rubythroat   ---------- - [361]
bluethroat            ---------- - [361]
budgerigar            SQVSPS*--- - [361]
blue_tit              S?-------- - [361]
great_tit             ---------- - [361]
marsh_tit             ---------- - [361]
Humboldts_penguin     S--------- - [361]
common_canary         SQVGPGQPRM * [361]
zebra_finch           SQVGPS*--- - [361]
tiger_salamander      SQVSPS*--- - [361]
firebelly_newt        SQVSPN*--- - [361]
American_bullfrog     SQVSPSAS*- - [361]
African_clawed_frog   SQVSPA*--- - [361]
```
